# Impact of guideline awareness on the counseling of patients with acute cough among general practitioners and pharmacy personnel

**DOI:** 10.1371/journal.pone.0254086

**Published:** 2021-08-05

**Authors:** Peter Kardos, Kai-Michael Beeh, Ulrike Sent, Guido Bissmann

**Affiliations:** 1 Group Practice, Center for Allergy, Respiratory and Sleep Medicine, Red Cross Maingau Hospital, Frankfurt am Main, Germany; 2 INSAF, Respiratory Research Institute, Wiesbaden, Germany; 3 Medical Affairs Consumer Healthcare, Sanofi-Aventis Deutschland GmbH, Frankfurt-Hoechst, Germany; Universidad de Antioquia, COLOMBIA

## Abstract

**Objective:**

To explore the awareness and knowledge of applicable guidelines on acute cough among general practitioners, pharmacists and pharmacy technicians and to compare their recommendation behavior and clinical decision making to the evidence-based recommendation in the applicable guidelines.

**Methods:**

An anonymous online survey was performed among 303 members of an existing panel of healthcare professionals (HCPs). They were presented with a hypothetical case vignette representative of their daily practice and asked for their treatment recommendations. After being shown an excerpt from the applicable guidelines, these questions were repeated.

**Results:**

Forty-six % of participants reported to seek information on cough and respiratory conditions very often or often. Among 12 non-prescription treatments-commonly used over-the-counter-products for acute cough, HCPs most often recommended various plant extract-based products (phytotherapeutic remedies) for the acute cough case, whereas chemically defined options such as ambroxol or N-acetyl-cysteine were recommended less often. Following presentation of the guidelines excerpt, recommendations of the phytotherapeutic remedies decreased moderately whereas that of the guideline-recommended ambroxol more than doubled. Among stated reasons for the recommendation guideline conformity increased from 5% to 35% among the top-3 reasons.

**Conclusions:**

The recommendations for the treatment of acute cough by professionals involved in primary healthcare deviated considerably from the applicable guideline recommendation but changed after presentation of a guidelines excerpt and knowledge thereof. We conclude that dissemination of applicable guideline knowledge is relevant to improve evidence-based healthcare and clinical decision making.

## Introduction

Cough is a physiological defense mechanism to assist the clearing of the airways from excess secretion and foreign materials; while this is beneficial to the individual, it can be disadvantageous to those around him due to promoting the spread of infections [1]. Cough is one of the most frequent symptoms leading to healthcare-seeking behavior, thereby leading to considerable healthcare expenditure [2] and the most frequent reason of acute cough is a viral airway infection [3] frequently concurrent with the common cold. Despite being a defense mechanism, cough is unpleasant and negatively affects sleep and overall well-being. Accordingly, many patients with acute cough seek medical help to mitigate symptoms and reduce their duration despite the typically self-limiting nature of the condition.

A recent local guideline of the German Respiratory Society has comprehensively analyzed the evidence on epidemiology, pathophysiology, diagnostic approaches and treatment of cough [3]. Further emphasis of the guideline is the physiology of cough in anticipation of the introduction of new drugs, as well as detailed treatments on cough triggered by affections in the upper respiratory tract. A classification via three new, simplified algorithms for acute, subacute and chronic cough is given. This guideline concludes that acute cough is suitable for self-management by the patient, e.g. using over-the-counter (OTC) medications, and does not warrant additional diagnostic investigations if warning signs are absent such as shortness of breath, hemoptysis, chest pain, high fever or evidence of pneumonia. Accordingly, most patients with acute cough are advised by pharmacy personnel such as pharmacists (PAs) and pharmacy technicians (PTs), but a sizeable fraction also by general practitioners (GPs). Public health requires that the advice given by these groups of healthcare professionals (HCPs) is correct, in the sense of evidence-based information tailored to the individual patient needs. This is particularly important because despite acute cough most often being caused by a viral airway infection, rare but serious causes of acute cough include serious or even life-threatening diseases such as pulmonary emboli, foreign body aspiration or spontaneous pneumothorax. This emphasizes the role of guidelines to facilitate evidence-based management of patients with acute cough.

Studies in various other indications indicate that guideline knowledge and adherence among physicians is suboptimal, for instance among primary care physicians [4] and pediatricians [5, 6] in functional constipation in children, urologists in prostate cancer screening [7], hospital-based physicians in infection management [8], or primary care physicians in the use of antibiotics in the management of acute lower respiratory tract infections [9]. Against this background, we have performed an online survey among groups of HCPs, specifically GPs, PAs and PTs to explore their awareness and knowledge of applicable guidelines on acute cough and to compare their recommendation behavior to that in the guideline; moreover, we have investigated whether recommendation behavior changes acutely following exposure to excerpts from the German Respiratory Society guideline [3]. A secondary aim of the survey was to compare these parameters between GPs, PAs and PTs.

## Methods

We have conducted an anonymous online survey between 27.8.-23.9.2019. The executing institute is DocCheck Research (www.research.doccheck.com) using a randomized sample out of the DocCheck panel. Anonymity was secured for the participants, the authors and the sponsor, but the intermediary DocCheck Research has information on the identity of the participants. The sample for the survey consisted of 303 HCPs, including 102 office-based GPs, 101 public pharmacy-based PAs and 100 public pharmacy-based PTs. This sample size was not based on formal calculations but on advice from the platform provider and previous experience with a similar survey in the area of constipation [10]. The panel members had given informed consent to participate; ethical committee approval or registration in a clinical trial registry were not applicable based on the anonymous nature of the survey and the lack of capturing clinical data from the participants. Additional information on survey design and conduct is presented in the S1 Data.

The self-administered survey initially asked how often (rating scale from 1 = never to 5 = very frequent) participants looked for information on cough and bronchial disease and which source of information were used. Thereafter, they were presented with a hypothetical case typical for their practice. Case 1 was presented to GPs and read “A 60-year-old male patient an acute cough suffers from a **diagnosed acute bronchitis caused by a virus**. The medical history and examination reveal no additional warning signs. The patient asks for a symptomatic therapy to relieve the intensity of his acute cough and to shorten its duration” (emphasis as originally shown to participants; the verbatim German text of both cases is shown in the S2 Data). Case 2 was presented to PAs and PTs and read “A 42-year-old female patient complains from an **acute cough** associated with a **common cold** with the typical range of symptoms and no further warning signs. There are no underlying medical conditions. The patient comes to you and asks for advice”. Thereafter, the participants were requested to rank 12 therapeutic options commonly used in Germany as recommended OTC treatments. These were ambroxol (e.g. Mucosolvan^®^ or various generics), cough remedy teas, demulcents (e.g. honey, cough candies), guaifenesin (e.g. Wick Hustenlöser^®^), ivy extract (e.g. Prospan^®^), myrtol (e.g. Gelomyrtol forte^®^), N-acetylcysteine (e.g. ACC akut^®^ or various other generics), pelargonium extract (e.g. Umckaloabo^®^), physiological saline/Emser salt, thyme & ivy extract (e.g. Bronchipret^®^), thyme & primrose extract (e.g. Bronchicum^®^), or “other” (free text option). To minimize bias, the sequence of presentation of these treatments on the list for ranking was randomized across participants. They were also asked to state the reasons behind the top-3 recommendations (free text), and to rank efficacy and tolerability against cough associated with common cold (PAs and PTs) or more specifically cough associated with common cold in a patient with acute viral bronchitis (GPs) for the 12 treatment options. Thereafter, they were asked about the strength of evidence for 7 treatment options (ambroxol, guaifenesin, ivy, myrtol, N-acetylcysteine, thyme & ivy and thyme & primrose) and to give a reason for the chosen top-3 options. Furthermore, they were asked to rate the importance of additional pharmacological properties of expectorants (e.g. anti-oxidative, anti-inflammatory, local anesthetic, anti-viral) a 5-point-Likert scale from not required (1) at all to extremely important (5).

The questionnaire then switched to the topic of guidelines asking how aware participants were about applicable guidelines on a scale from 1 (not aware at all) to 5 (very well aware). This was extended by a yes/no question on the awareness of specific applicable guidelines, i.e. from Bundesvereinigung Deutscher Apothekerverbände e.V. and Bundesapothekerkammer (BAK) [11], guidelines of the Association of the Scientific Medical Societies (AWMF) such as the German Respiratory Society [3], and the German College of General Practitioners and Family Physicians (DEGAM) [12]. After these questions, participants were presented with an excerpt for the 2019 cough guidelines of the German Respiratory Society [3] (verbatim German text of excerpt and English translation shown in S2 Data). Participants were asked whether the content of the new guideline was known to them (options: yes, fully; yes, partly; no, unknown). Thereafter, the questions related to the original vignette case were repeated, now in light of the guideline excerpts just presented. The survey concluded by capturing some demographic variables including age, gender and size of the city where the pharmacy or GP office was located. The original text of the survey and an English translation are presented as S2 Data.

Data are shown as absolute numbers or as % of responders; several of the questions allowed to concomitantly choose multiple options. In the ranking questions, % of responders chosen an item as part of top-3 recommendations (if 12 options given) or top-2 recommendations (if 7 or less options given) are presented. Based on the exploratory character of the survey and in line with recent guidelines for performing and reporting statistical analysis [13], no hypothesis-testing statistical analysis was performed and all data are reported in a descriptive manner only.

## Results

### General information

A total 303 HCPs participated in the survey (102 office-based GPs and 101 PAs and 100 PTs). 66% were female (36% among GPs, 65% among PAs and 96% among PTs), and 54% were under 45 years of age and 23% 45–65 years of age (under 45 years 28% of GPs, 53% of PAs and 80% of PTs). They covered all types of communities from small towns with less than 5000 to large cities with more than 1 million inhabitants and provided a reasonable representation of all areas in Germany.

Participant self-assessment on how often they seek information on cough and bronchial conditions is shown in Table 1. Across all groups very often and often was reported by 46% but was highest among GPs (53%) and lowest among PTs (40%). The most frequently reported sources of information were journals (82%), training offered by pharmaceutical manufacturers (55%), training offered by medical or pharmaceutical councils (49%), treatment guidelines (48%), online information sources for HCPs (46%), other online sources (40%), publications by medical associations (32%), discussion with patients and customers (24%), training offered by professional associations (20%), reports on TV or radio (4%) and other sources (5%; multiple nominations possible). While patterns of used information sources were generally similar between groups of participants, some quantitative differences emerged: GPs more often reported to consult guidelines (68% vs. 47% and 30% by PAs and PTs, respectively), publications by medical associations (53% vs. 23% and 21%) and lectures by professional associations (32% vs. 25% and 11%), whereas PAs and PTs more often used training offered by pharmaceutical manufacturers (62% and 76% vs. 26%), training offered by medical or pharmaceutical councils (62% and 52% vs. 32%) and training offered by medical associations (44% and 37% vs. 14%).

**Table 1:** Participant self-assessment on information seeking on cough and bronchial conditions; data are shown as % of responders (rounded to full number; based on 303 participants including 102 GPs, 101 PAs and 100 PTs) rating on a scale from 1 (never) to 5 (very frequent).

Rank	Total	GP	PA	PT
1	0	0	0	1
2	7	9	7	5
3	47	38	49	54
4	37	42	40	30
5	9	11	5	10

### Pre-guideline recommendations

When ranking the 12 treatment options based on efficacy, the most frequently named options as top-3 recommendations were in descending order thyme & ivy, myrtol, thyme & primrose, ivy, ambroxol and N-acetylcysteine (all others named by 15% of less). However, the ranking based on efficacy differed between professional groups: While GPs considered thyme & ivy, myrtol and thyme & primrose similarly often to be in the top-3 for efficacy (all 42–45%), PAs and PTs more often considered thyme & ivy (71–72%). When ranking the 12 treatment options based on tolerability, a different pattern emerged as the most frequently named options were in descending order thyme & ivy, physiological saline/Emser salt, ivy, cough remedy teas, thyme & primrose, demulcents and ambroxol (all others named by 12% or less). Perceptions of tolerability differed between types of HCPs with the highest rated treatments based on tolerability being physiological saline or Emser salt, cough remedy teas and ivy for GPs, thyme & ivy, physiological saline/Emser salt and thyme & primrose for PAs and thyme & ivy, ivy and equally often mentioned thyme & primrose and physiological saline or Emser salt by PTs.

**Fig 1:**
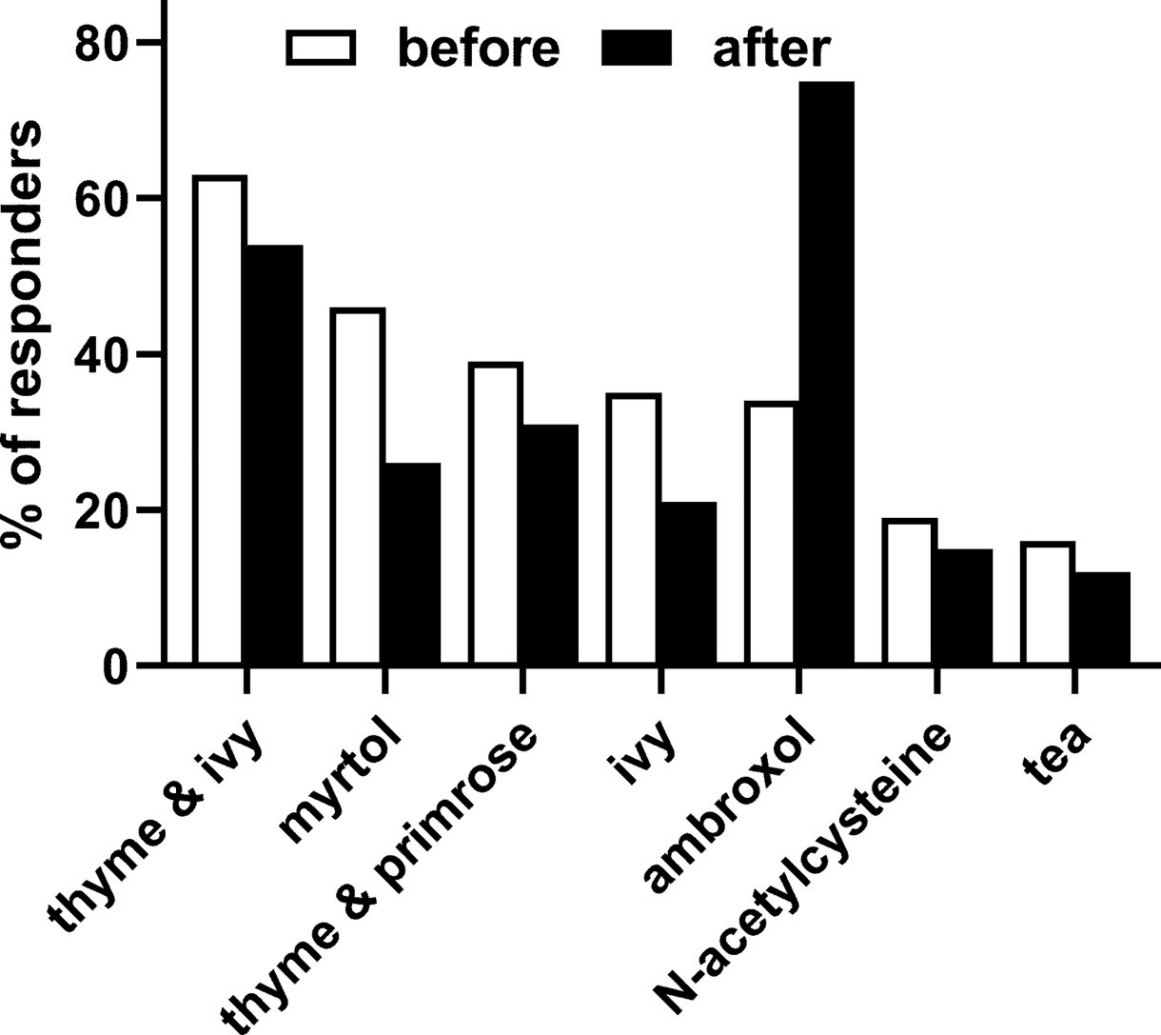
Seven most frequently recommended treatments for the presented case as reported before and after being shown excerpts of an applicable guideline. Data are shown as % of responders naming an option as part of top-3 recommendations. Only 7 most frequently named options are shown; for others see main text. Note that the typical case presented to GPs differed somewhat from that presented to pharmacy personnel.

After being presented with a typical case in daily consulting practice (as described in section Methods/S2 Data), the most often recommended treatments were thyme & ivy extract (60%), myrtol (49%), a thyme & primrose extract (38%), an ivy extract (37%), ambroxol (30%) or N-acetylcysteine (20%) (Fig 1). Other options such as cough remedy teas (16%), physiological saline or Emser salt (14%), pelargonium extract (11%), demulcents (6%), guaifenesin (2%) or others (4%) were recommended less often. While PAs and PTs recommended a thyme & ivy extract most often (71% and 70%) followed by myrtol (50% and 66%), GPs recommended thyme & ivy, thyme & primrose, ivy and myrtol similarly often (all 40–41%). Ambroxol was recommended more often by PAs and PTs than by GPs (36% and 35% vs. 21%), GPs and PAs recommended N-acetylcysteine more often than PTs (26% and 22% vs. 12%).

**Fig 2:**
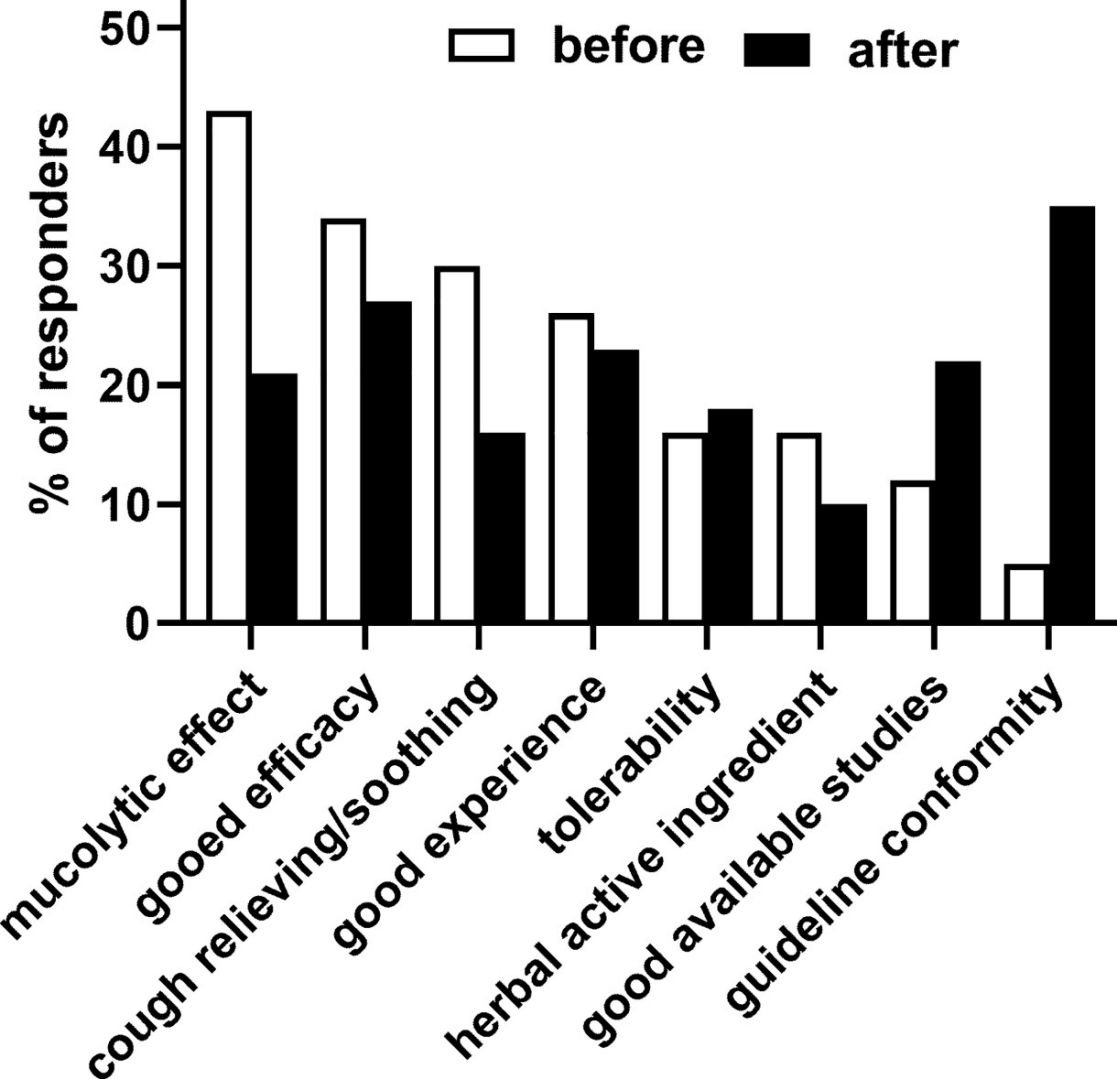
Stated reasons underlying the top-3 recommendation for the presented case as provided before and after having been shown excerpts of an applicable guideline. Data are shown as % of responders naming an option as part of top-3 recommendations. Note that the typical case presented to GPs differed somewhat from that presented to pharmacy personnel.

The most frequently reported reasons for the top-3 recommendations in descending order were mucolytic effect, good efficacy, relaxation/cough-relieving/soothing and good/own experience (Fig 2). Fewer participants named other reasons including broad spectrum of effects (15%), anti-inflammatory effects (13%), good available studies (12%), comfortable dosage form (11%), fluid supply (11%), patient/customer acceptance (7%), good combination of active ingredients (4%), anti-viral effects (5%) and guideline conformity (5%). While some reasons were similarly reported by all three groups of HCPs, some were preferentially considered. For instance, mucolytic effects were more often mentioned by PAs and PTs than by GPs (48% and 47% vs. 34%), good efficacy more often by GPs and PTs than by PAs (41% and 38% vs. 24%) or good/own experience more often by GPs and PAs than by PTs (32% and 31% vs. 19%).

**Fig 3:**
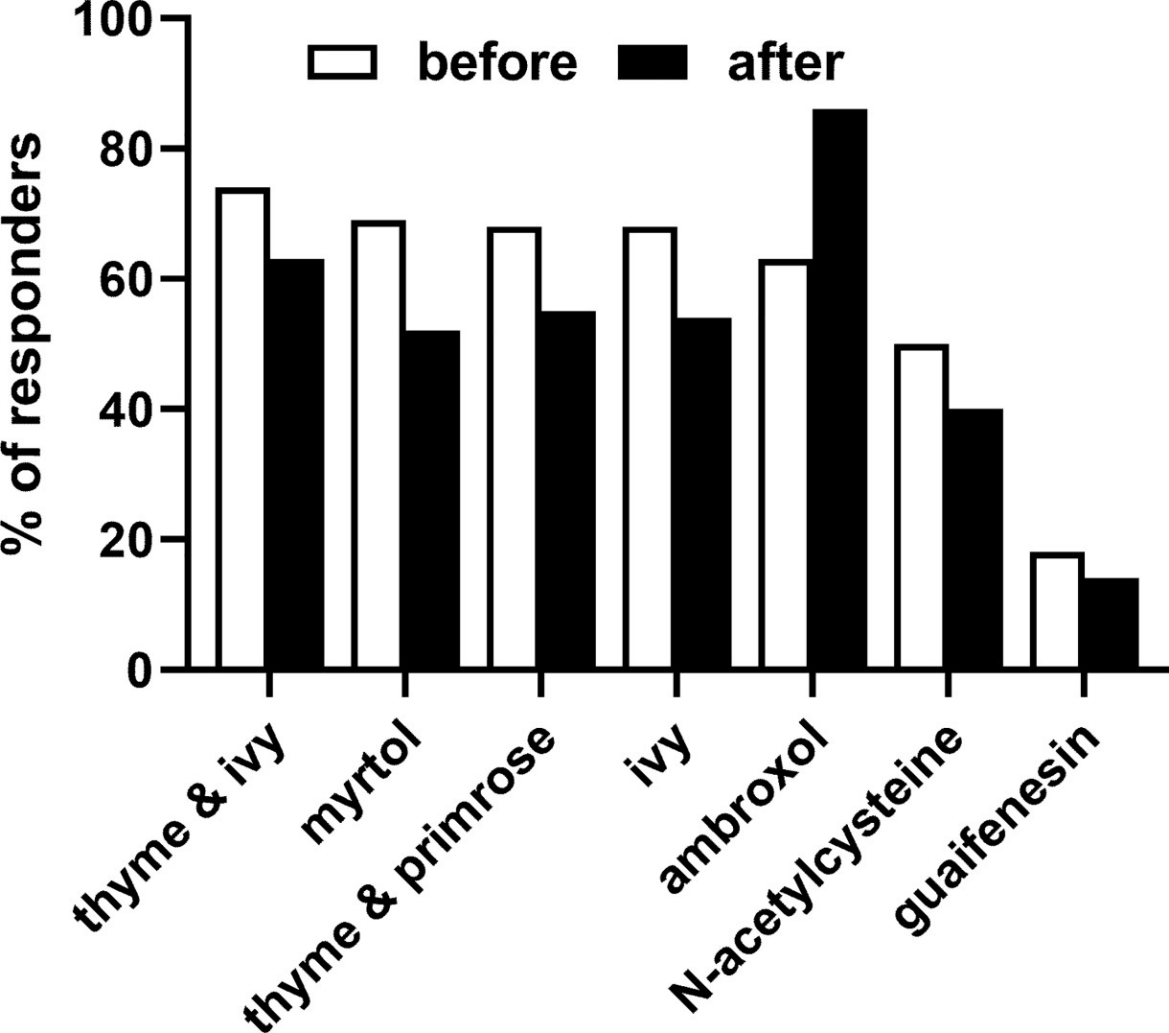
Perceived strength of evidence related to 7 selected anti-cough treatments as reported before and after being shown excerpts of an applicable guideline. Data are shown as % of responders rating strength of evidence as very good or good.

Participants ranked the strength of evidence supporting the use of 7 treatment options in descending order as thyme & ivy, myrtol, thyme & primrose, ivy, ambroxol, N-acetylcysteine and guaifenesin (Fig 3). While the rank order was comparable among HCP groups, PAs and PTs consistently considered the strength of evidence higher than physicians (GPs) (76–88% and 78–90% vs. 36–45% for a good to very good rating). Leading top-2 reasons behind strength of evidence perception were “good study situation” (57%), “proven effect” (33%), “good effectiveness” (19%), “experience” (19%) and “guideline recommendation (11%); a variety of other reasons was mentioned by <10% of participants. Reasons behind perceptions of strength of evidence were comparable among HCP groups.

Participants ranked the desirability of additional pharmacological properties of expectorants (anti-oxidative, anti-inflammatory, anti-viral, local anesthetic) on a scale from 1 (not required at all) to 5 (extremely required). A ranking of 4–5 was given to anti-inflammatory by 73%, anti-viral by 65%, anti-oxidant by 23% and local anesthetic by 21%. This assessment was similar among all three groups of HCPs.

### Post-guideline recommendations

The ranking of the 12 treatment options based on perceived efficacy changed after presentation of guideline excerpts [3]: Most treatment options declined in number of mentions in the top-3 recommendations, most strongly for myrtol (from 46% to 26%) and ivy (from 35% to 21%), whereas that of ambroxol increased considerably (from 34% to 75%). This pattern was similar among all three groups of HCPs. Similarly, the ranking of perceived tolerability changed with most options declining, most strongly for physiological saline or Emser salt (from 49% to 31%) and ivy (from 39% to 22%), whereas that of ambroxol increased from 18% to 57% as being part of the top-3 ranks. This pattern of change was comparable across all three groups of HCPs.

After presentation with excerpts from the applicable guideline, some recommendation behaviors changed (Fig 1): While the most frequently mentioned top-3 recommendations prior to showing the guideline excerpts all decreased (strongest decrease for myrtol and ivy), recommendation of ambroxol more than doubled (overall from 30% to 73%, GPs from 21% to 67%, PAs from 36% to 77% and PTs from 35% to 75%). This was associated with a change in reported underlying reasons for recommendations (Fig 2): While reasons for treatment recommendation such as mucolytic effect and relaxation/cough-relieving/soothing decreased by about half, good available studies about doubled and guideline conformity increased (overall from 5% to 35%, GPs from 2% to 32%, PAs from 9% to 43% and PTs from 4% to 29%).

The perceived strength of evidence changed in a similar way as the ranking in the top-2 recommendations with the list of 7 treatment options, although to a smaller extent (Fig 3). Thus, perceived strength of evidence declined for all options, most strongly for myrtol (from 69% to 52%), except for ambroxol which increased from 63% to 86%. Among reasons behind perceived strength of evidence, “good study situation” showed no major change, whereas “proven effect” declined from 33% to 17%, “good effectiveness” from 19% to 14%, and “experience” from 19% to 15%; in contrast “guideline recommendation” increased from 11% to 35%; all other reasons stayed below 10%. The pattern of stated reasons was comparable among groups of HCPs.

The perceived desirability of additional pharmacological properties of expectorants (irrespective of specific compounds/extracts) changed only slightly, with similar changes across all three groups of HCPs.

### Self-assessment of guideline knowledge

In a self-assessment of familiarity with applicable guidelines on a scale of 1 (not familiar at all) to 5 (very familiar) participants rated their knowledge as 1, 2, 3, 4 and 5 in 5%, 16%, 45%, 30% and 4% of cases, respectively. These ratings were comparable across professional groups. Participants stated to be most familiar with two applicable guidelines from the AWMF (54%) and the German Chamber of Pharmacists (41%), and less so with those of the German Association of Pharmacists (31%) or European medical associations such as the European Respiratory Society (15%). As could be expected, there were marked differences between professional groups with 87% of GPs reporting awareness of guidelines by AWMF (48% and 28% for PAs and PTs), whereas PAs and PTs reported preferential familiarity with those from the Federal Chamber of Pharmacists (63% and 59%) and that of the German Association of Pharmacists (45% and 48%), both being largely unknown to GPs. European guidelines were most often perceived as familiar to GPs (23%) and less so to PAs (13%) and PTs (8%). Thus, different professional groups involved in the primary care of patients with cough preferentially are familiar with distinct guidelines targeted at their profession. After being presented with excerpts of the most recent applicable acute cough guideline [3], 4% of participants claimed to have been fully familiar with all of the content, 55% to be partly familiar with the content and 40% realized that they had not been previously aware of the content, indicating that many participants over-estimated their familiarity. This pattern was similar with GPs and PAs, but partly familiarity and lack of awareness of content was higher among PTs (41% and 58%, respectively).

## Discussion

Our results indicate that HCPs frequently over-estimate their familiarity with an applicable guideline; however, upon exposure to guideline-based information they adapt their recommendations. Guidelines are a general approach to harmonize behavior. When issued by authoritative bodies such as learned societies, they can improve patient care by assisting the implementation of evidence-based medicine. Accordingly, many societies in the field of respiratory medicine have issued guidelines for the diagnosis and management of cough such as the American College of Chest Physicians [14], the Chest Expert Panel report on acute cough [15], the British Thoracic Society [16] and the European Respiratory Society [17]. The German Respiratory Society has initially issued guidelines for the diagnosis and treatment of cough in adults in 2004 and reported the second major update in early 2019 [3] including chapters on epidemiology, (patho) physiology, classification and diagnostic / treatment approaches to reflect the current state of knowledge on acute cough in adults. Against this background, our survey was designed to explore the knowledge and recommendation/prescription behavior of HCPs involved in primary care of patients with acute cough and the acute impact of showing excerpts from an applicable guideline on knowledge and recommendations. As a secondary aim we compared these parameters between GPs, PAs and PTs.

### Critique of methods

Our analyses are based on an existing panel of HCPs that had registered interest in participating in surveys related to their area of expertise. Therefore, they cannot necessarily be assumed to be representative for all HCPs involved in the primary care of patients with acute cough and may, if anything, have an above-average state of interest and knowledge in this area. The rating scales supplied to the participants lead to somewhat subjective responses. This was intentional as we wished to capture awareness and knowledge of the participants. Based on the exploratory nature of the study and recent recommendations related to statistical analysis [13], we had chosen to present descriptive data only and to focus on effect sizes, but not to perform statistical analyses.

Several organizations within Germany [3, 11, 12] and internationally [14–17] have issued guidelines on the diagnosis and treatment of acute cough. The specific recommendations in these guidelines differ to some extent. These differences may represent various factors including availability and/or popularity of some treatments in a given region, the target group of the guideline for instance primary vs. specialist care or physicians vs. pharmacists and the date when such guidelines have been issued. We have chosen the guidelines from the German Respiratory Society [3] as yardstick for our survey because it is the most recent and most comprehensive in Germany. While primarily intended for pulmonologists, this guideline has also widely been communicated to the primary care community especially for the treatment of acute cough, several months before our survey was performed, for instance in the Deutsche Apotheker Zeitung, a German weekly for pharmacists [18]. These considerations should be taken into account in the interpretation of our findings.

### Guideline awareness

According to our survey, the self-assessment of familiarity with applicable guidelines in the field of acute cough was limited (34% rating it has familiar to very familiar). After presentation with an excerpt from the guideline 4%, 55% and 40% claimed to have been fully, partially or not at all aware of it. This observation is in line with that of a recent indication-overarching survey among 1068 professionals working in public pharmacies in Germany, which found that 83% considered clinical trial data as important for the counseling of customers considering to buy OTC products but only 48% reported to base their recommendations in most to almost all consultations on clinical trial data; 69% reported difficulties in including clinical trial data in their counseling and only 7% claimed to read clinical trial data at least once a month [19].

The applicable guidelines recommends the symptomatic treatment of acute cough with expectorants, i.e. phytotherapeutics or ambroxol for which efficacy has been proven to mitigate symptoms and shorten the duration of the condition [3]. According to the guidelines, phytotherapeutics drugs with proven efficacy against cough include those from ivy [20], cineole [21], myrtol [22, 23], Pelargonium sidoides [24] and combinations of thyme & ivy [25] and thyme & primrose [26]. The only chemically defined expectorant available in Germany with proven efficacy in clinical studies is ambroxol [22]. In contrast, little evidence supports the use of extracts from Papaver somniferum or Ephedra sinica despite their century long use in traditional medicine [3]. The recommendation behavior of participants in our survey was only partly in line with the evidence-based guidelines: 5 of the 6 most often recommended treatments were guideline-endorsed, but one (N-acetylcysteine) was not. Moreover, despite a similar degree of endorsement by the guideline, some (thyme & ivy) were recommended by 60% whereas others (ambroxol) were recommended by 30%. One guideline-endorsed treatment (pelargonium) was less frequently recommended than three others that were not guideline endorsed due to a lack of evidence from controlled clinical trials. A similar pattern of only partial guideline adherence was observed for perceived efficacy and tolerability and perceived strength of evidence. These data are in line with the self-assessment of survey participants related to familiarity with the guideline.

Following exposure to an excerpt from the applicable guidelines [3], recommendation behavior by the survey participants changed. Most notably, the share of ambroxol recommendations (the only chemically defined expectorant with proven efficacy according to the guideline) increased markedly, leading to a reduction in the share of recommendations for all other treatments. A similar pattern was observed for perceived efficacy, perceived tolerability and, to a smaller degree, perceived strength of evidence. In addition, ‘guideline conformity’ shifted from a rarely mentioned top-3 reason for recommendation to the most frequently mentioned one. These data show that acute exposure to applicable guideline content and thus enhanced knowledge can affect recommendation behavior. However, this adaptation was only partial as it did not affect the recommendation ratings of some guideline-recommended treatments (pelargonium) that were not explicitly mentioned in the excerpts, whereas other treatments not endorsed in the guideline due to lack of evidence remained being recommended (N-acetylcysteine or anti-cough teas). Moreover, some HCPs rated the strength of evidence highly for some treatments (N-acetylcysteine or guaifenesin) for which the guideline states a lack of evidence. The fact that these treatments were not explicitly mentioned in the excerpts (which were verbatim from the guidelines) further highlights that guideline awareness can change recommendation behavior.

The applicable guidelines state that self-management of acute cough should be limited to cases where warning signs such as shortness of breath, hemoptysis, chest pain, higher fever, evidence of pneumonia have been excluded [3]. Whether adherence to this part of the guidelines is better than that related to specific treatment recommendations has not been studied in our survey. Based on the observed deviations from the guidelines for treatment recommendations, we consider that future studies should explore adherence to diagnostic recommendations in the interest of public health.

### Differences between groups of HCPs

A secondary aim of the study was to compare recommendation behavior and perceptions by groups of HCPs. While we have not performed a formal statistical analysis of group differences based on the exploratory nature of our study, several group differences of considerable magnitude were observed. Firstly, groups of participants preferred different sources of information. For instance, pharmacy-based HCPs more often reported using training by pharmaceutical manufacturers (e.g., sponsored symposia) and less often treatment guidelines as compared to GPs. When using guidelines, physicians more often used those issues by medical associations, whereas PAs and PTs more often used those issued by pharmacist associations. Overall, PTs reported the least familiarity with guidelines. The recommendations made by PAs and PTs and their perception of strength of underlying evidence also differed at least for some treatments from those by GPs, for instance recommendation of thyme & ivy by 70–71% of PAs and PTs as compared to 40% by GPs.

While these examples suggest that differences between groups of HCPs may largely reflect those between physicians and pharmacy-based HCPs, these professional differences are clearly not the only ones. For instance, GPs and PAs were closer to each other in their recommendation behavior related to use of N-acetylcysteine or related to the role of experience in making treatment recommendations than each of them compared to PTs. Interestingly, differences in recommendations and underlying perceptions and reasoning between groups of HCPs became smaller after exposure to guideline excerpts. This heterogeneity between groups of HCPs further supports the view that recommendation behavior and perceptions of efficacy, tolerability and strength of evidence are at least partly based on factors (e.g. lack of knowledge) other than available evidence as summarized in the applicable guidelines [3].

## Conclusions

The rationale use of healthcare resources requires that treatment approaches are evidence-based. Given that acute cough is both one of the most frequent cause for patients seeking advice from a HCP [2] and the most bothering and long lasting symptom e of acute bronchitis/common cold, we found it surprising that less than 40% of HCPs self-report to be familiar or very familiar with the contents of guidelines related to the treatment of cough. The self-assessment of familiarity with the contents of the applicable guidelines after being confronted with excerpts of it shows that the initial self-assessment may even have been over-optimistic. Accordingly, treatment recommendations and perceptions of efficacy and tolerability and of strength of underlying evidence deviate from the applicable guidelines. GPs, PAs and PTs apparently have quantitatively different recommendation behavior, reasoning behind their recommendations and perceptions of efficacy and tolerability, further indicating that many of those opinions and perceptions are rather empirical than evidence based. However, our data also show that presentation of guideline excerpts and thus knowledge of guideline recommendations acutely changes treatment recommendation behavior when counselling for OTC drugs. While it remains to be tested whether such changes are long-lasting, these findings suggest that greater efforts are needed to implement applicable guidelines to HCPs.

## Supporting information

S1 Data Additional information on survey design and conduct. (DOCX)

S2 Data Full text of questionnaire in its original German version and an English translation thereof. (DOCX)

## List of abbreviations

GP: general practitioner
HCP: healthcare professional
OTC: over the counter
PA: pharmacist
PT: pharmacy technician

## References

[1] Irwin RS. Introduction to the diagnosis and management of cough: ACCP evidence-based clinical practice guidelines. Chest. 2006;129(1):25S–7S. doi: 10.1378/chest.129.1_suppl.25S 16428688

[2] Morice A, Kardos P. Comprehensive evidence-based review on European antitussives. BMJ Open Respiratory Research. 2016;3(1):e000137. doi: 10.1136/bmjresp-2016-000137 27547407 PMC4985807

[3] Kardos P, Dinh QT, Fuchs KH, Gillissen A, Klimek L, Koehler M, et al. Leitlinie der Deutschen Gesellschaft für Pneumologie und Beatmungsmedizin zur Diagnostik und Therapie von erwachsenen Patienten mit Husten. Pneumologie. 2019;73(3):143–80. Epub 18.02.2019. doi: 10.1055/a-0808-7409 30776835

[4] Barnes J, Coleman B, Hwang S, Stolic A, Bousvaros A, Nurko S, et al. Educational needs in the diagnosis and management of pediatric functional constipation: a US survey of specialist and primary care clinicians. Postgrad Med. 2018;130(4):428–35. doi: 10.1080/00325481.2018.1464364 29667860

[5] Yang CH, Punati J. Practice patterns of pediatricians and trainees for the management of functional constipation compared with 2006 NASPGHAN guidelines. J Pediatr Gastroenterol Nutr. 2015;60(3):308–11. doi: 10.1097/MPG.000000000000059100005176-201503000-00010 25714574

[6] Scarpato E, Quitadamo P, Roman E, Jojkic-Pavkov D, Kolacek S, Papadopoulou A, et al. Functional gastrointestinal disorders in children: a survey on clinical approach in the Mediterranean area. J Pediatr Gastroenterol Nutr. 2017;64(6):e142–e6. doi: 10.1097/MPG.0000000000001550 28541259

[7] Tiedje D, Quer O, Breil B, Schrader AJ, Bothe C, Kruse K, et al. Anwendung der S3-Leitlinie zur Prostatakrebsfrüherkennung in urologischen Praxen. Der Urologe. 2017;56(7):910–6. doi: 10.1007/s00120-017-0352-1 28280863

[8] McKenzie KE, Mayorga ME, Miller KE, Singh N, Arnold RC, Romero-Brufau S. Notice to comply: a systematic review of clinician compliance with guidelines surrounding acute hospital-based infection management. Am J Infect Control. 2020;in press. doi: 10.1016/j.ajic.2020.02.006 32192754

[9] Kraus EM, Pelzl S, Szecsenyi J, Laux G. Antibiotic prescribing for acute lower respiratory tract infections (LRTI)–guideline adherence in the German primary care setting: An analysis of routine data. PLoS One. 2017;12(3):e0174584. doi: 10.1371/journal.pone.0174584 28350820 PMC5370139

[10] Eberlin M, Landes S, Biber-Feiter D, Michel MC. Impact of guideline awareness in public pharmacies on counseling of patients with acute or chronic constipation in a survey of pharmacy personnel. BMC Gastroenterol. 2020;20(1):191. doi: 10.1186/s12876-020-01338-4 32552767 PMC7301513

[11] Anonymous. ABDA/ BAK Leitlinie Husten 2019 [cited 2020 1.4.2020]. Available from: https://www.abda.de/fuer-apotheker/qualitaetssicherung/leitlinien/leitlinien-und-arbeitshilfen/.

[12] Heintze C, Krüger S, Gehrke-Beck S, Holzinger F. Husten. DEGAM-Leitlinie Nr. 11: Deutsche Gesellschaft für Allgemeinmedizin und Familienmedizin,; 2014 [cited 2020 19.3.2020]. Available from: https://www.degam.de/files/Inhalte/Leitlinien-Inhalte/Dokumente/DEGAM-S3-Leitlinien/Leitlinien-Entwuerfe/053-013_Husten/Langfassung_Leitlinie_Husten_20140323.pdf.

[13] Michel MC, Murphy TJ, Motulsky HJ. New author guidelines for displaying data and reporting data analysis and statistical methods in experimental biology. Mol Pharmacol. 2020;97(1):49–60. doi: 10.1124/mol.119.118927 31882404

[14] Irwin RS, Baumann MH, Bolser DC, Boulet L-P, Braman SS, Brightling CE, et al. Diagnosis and management of cough executive summary: ACCP evidence-based clinical practice guidelines. Chest. 2006;129(1):1S–23S. doi: 10.1378/chest.129.1_suppl.1S 16428686 PMC3345522

[15] Malesker MA, Callahan-Lyon P, Ireland B, Irwin RS, Adams TM, Altman KW, et al. Pharmacologic and nonpharmacologic treatment for acute cough associated with the common cold: CHEST Expert Panel Report. Chest. 2017;152(5):1021–37. doi: 10.1016/j.chest.2017.08.009 28837801 PMC6026258

[16] Morice AH, McGarvey L, Pavord I. Recommendations for the management of cough in adults. Thorax. 2006;61(suppl 1):i1–i24. doi: 10.1136/thx.2006.065144 16936230 PMC2080754

[17] Morice AH, Millqvist E, Bieksiene K, Birring SS, Dicpinigaitis P, Domingo Ribas C, et al. ERS guidelines on the diagnosis and treatment of chronic cough in adults and children. Eur Respir J. 2020;55(1):1901136. doi: 10.1183/13993003.01136-2019 31515408 PMC6942543

[18] Anonymous. Trocken oder verschleimt? Bei Husten nicht mehr die Frage: Deutscher Apotheker Verlag; 2019 [updated 23.4.201921.3.2020]. Available from: https://www.deutsche-apotheker-zeitung.de/news/artikel/2019/04/23/trocken-oder-verschleimt-bei-husten-nicht-mehr-die-frage/chapter:all%5b24.04.2019.

[19] Moritz K, Seiberth JM, Schiek S, Bertsche T. The impact of evidence from clinical trials on counselling for over-the-counter drugs: A national survey of pharmaceutical staff in German pharmacies. J Clin Pharm Ther. 2019;44(6):895–903. doi: 10.1111/jcpt.13013 31479521

[20] Schaefer A, Kehr MS, Giannetti BM, Bulitta M, Staiger C. A randomized, controlled, double-blind, multi-center trial to evaluate the efficacy and safety of a liqid containing ivy leaves dry extract (EA 575R) vs. placebo in the tretament of adults with acute cough. Pharmaczie. 2016;71(9):504–9. doi: 10.1691/ph.2016.6712 29441845

[21] Fischer J, Dethlefsen U. Efficacy of cineole in patients suffering from acute bronchitis: a placebo-controlled double-blind trial. Cough. 2013;9(1):25. doi: 10.1186/1745-9974-9-25 24261680 PMC3842692

[22] Matthys H, De Mey C, Carls C, Rys A, Geib A, Wittig T. Efficacy and tolerability of myrtol standardized in acute bronchitis. A multi-centre, randomised, double-blind, placebo-controlled parallel group trial vs. cefuroxime and ambroxol. Arzneimittelforschung. 2000;50(8):700–11. doi: 10.1055/s-0031-1300276 10994153

[23] Gillissen A, Wittig T, Ehmen M, Krezdorn HG, de Mey C. A muliti-centre, randomised, double-blind, placebo-controlled clinical trial on the efficacy and tolerability of GeloMyrtolR forte in acute bronchitis. Drug Res. 2013;63(1):19–27. doi: 10.1055/s-0032-1331182 23447044

[24] Timmer A, Günther J, Motschall E, Rücker G, Antes G, Kern WV. Pelargonium sidoides extract for treating acute respiratory tract infections. Cochrane Database of Systematic Reviews. 2013;22(10):CD006323. doi: 10.1002/14651858.CD006323.pub3 24146345 PMC11835051

[25] Kemmerich B, Eberhardt R, Stammer H. Efficacy and tolerability of a fluid extract combination of thyme herb and ivy leaves and matched placebo in adults suffering from acute bronchitis with productive cough. A prospective, double-blind, placebo-controlled clinical trial. Arzneimittelforschung. 2006;56(9):652–60. doi: 10.1055/s-0031-1296767 17063641

[26] Kemmerich B. Evaluation of efficacy and tolerability of a fixed combination of dry extracts of thyme herb and primrose root in adults suffering from acute bronchitis with productive cough. A prospective, double-blind, placebo-controlled multicentre clinical trial. Arzneimittelforschung. 2007;57(9):607–15. doi: 10.1055/s-0031-1296656 17966760

