# Supplementary material for: Impact of guideline awareness on the counseling of patients with acute cough among general practitioners and pharmacy personnel
Source: PLoS One. 2021 Aug 5;16(8):e0254086. doi: 10.1371/journal.pone.0254086 (PMC8341580; doi:10.1371/journal.pone.0254086)
Supplement: S1 Data — (DOCX) [file pone.0254086.s001.docx]

**S1 Data**

**Impact of guideline awareness on the counseling of patients with acute cough among general practitioners and pharmacy personnel**

Peter Kardos, Kai-Michael Beeh, Ulrike Sent; Guido Bissmann

Additional information on survey design and conduct

The DocCheck Research platform works as follows: The provider has an existing panel of various HCP groups that have indicated their willingness to participate in anonymous surveys. When a new survey is launched, an invitation mail goes out to all panel members in a specific group of HCPs (e.g., GPs). The survey closes when the a priori specified required number has participated (sometimes multiple people are simultaneous actively filling the survey, so that the final number is closed after those have completed, resulting in a sample size of slightly more than 100 in a group).

Calculation of responder rate is typical for a classic survey but based on these technical aspects of the platform being used does not make sense.

The current numbers of participants are not based on formal sample size calculations. Our statistics consultant had informed us that formal sample size calculations require estimates on effect sizes and variability, neither is applicable to the present exploratory work. Therefore, sample sizes were chosen based on advice from the platform provider (DocCheck Research) and on prior experience with a similar survey related to guideline awareness in self-medication of constipation.

The 303 participants came from the following German federal states (“Länder”; data as % rounded to full numbers): Baden-Württemberg 15, Bavaria 16, Berlin 7, Brandenburg 2, Bremen 0, Hamburg 2, Hessen 9, Mecklenburg-West Pomerania 1, Lower Saxony 9, North Rhine-Westphalia 21, Rhineland-Palatinate 2, Saarland 3, Saxony 4, Saxony-Anhalt 4, Schleswig-Holstein 4, Thuringia 2. This is reasonably balanced within Germany, for instance the three Länder with the highest number of participants also are those with the largest populations (Baden-Württemberg, Bavaria and North Rhine-Westphalia), whereas Länder with small populations had only few participants (e.g., Bremen, Brandenburg and Saarland). This is explained at the end of the 1^st^ paragraph of Results.

The survey included multiple types of questions, including multiple choice with a single or with multiple possible answers, 5-point-Likert scales or questions in which participants were asked to rank options in drop & drag fashion in the online version.

The survey was developed by the authors. It went online on 27.8.2019 and closed (target number of participants reached) on 23.9.2019. Time between finalizing the survey questionnaire (6.8.2019) and start of survey primarily reflects the administrative work by the provider to let the survey go live. We hope that this addresses the specific questions of the reviewer.
