# Supplementary material for: Impact of guideline awareness on the counseling of patients with acute cough among general practitioners and pharmacy personnel
Source: PLoS One. 2021 Aug 5;16(8):e0254086. doi: 10.1371/journal.pone.0254086 (PMC8341580; doi:10.1371/journal.pone.0254086)
Supplement: S2 Data — (DOCX) [file pone.0254086.s002.docx]

**S2 Data**

**Impact of guideline awareness on the counseling of patients with acute cough among general practitioners and pharmacy personnel**

Peter Kardos, Kai-Michael Beeh, Ulrike Sent; Guido Bissmann

Original questionnaire presented to German survey participants

(English translation hereunder)

**F1. Informationsverhalten**

Zunächst geht es um das Thema Fachinformationen.
Ganz generell: Wie häufig informieren Sie sich speziell zum **Thema Husten/Bronchialerkrankungen**?

*Skala: nie | selten | gelegentlich | häufig | sehr häufig*

**F2. Informationsquellen**

Welche Quellen nutzen Sie in der Regel, um Informationen zum **Thema** **Husten/Bronchialerkrankungen** zu erhalten?

*Bitte wählen Sie alle Antworten, die für Sie zutreffen.*

1. Publikationen der medizinischen Fachgesellschaften
2. Fachzeitschriften
3. Therapie-Leitlinien
4. Fachinformationen im Internet
5. Gespräche mit dem Außendienst
6. Trainings/Schulungen von Pharmaherstellern
7. Trainings/Schulungen von Kammern
8. Fortbildungen der Apotheker-/Ärztekammer
9. Trainings/Schulungen von Verbänden
10. Fachvorträge Verbände
11. Berichte im TV, Radio
12. Gespräche mit Kunden/Patienten
13. Internet (z.B. Google/ Wikipedia)
14. Sonstiges:_______________

**FALLBEISPIEL 1 (F3-F6) NUR AN APOTHEKER/PTA**

Im Folgenden geht es um das Thema **akuter Erkältungshusten**, speziell um die Bewertung ausgewählter rezeptfreier Expektorantien zur Behandlung von akutem Husten.

**Folgendes Fallbeispiel:**

Eine 42-jährige Kundin klagt über **akuten Husten** bedingt durch einen **Erkältungsinfekt** mit typischer Symptomatologie ohne weitere Alarmzeichen. Es liegt keine Grunderkrankung vor. Diese Kundin kommt zu Ihnen und sucht Rat.

Beantworten Sie für dieses Fallbeispiel die nachfolgenden Fragen.

**F3. Produktempfehlung**

Hier sehen Sie verschiedene Wirkstoffe/Produkte für die Behandlung eines **akuten Erkältungshusten.**

Welche dieser Wirkstoffe/Produkte **empfehlen** Sie zur Linderung des Hustens der Kundin aus dem eben beschriebenen Fallbeispiel bevorzugt?

1. N-Acetylcystein (z.B. ACC akut, ACC Generika)
2. Ambroxol (z.B. Mucosolvan, Ambroxol Generika)
3. Thymian/Primel (z.B. Bronchicum)
4. Efeu (z.B. Prospan)
5. Guaifenesin (z.B. WickHustenlöser)
6. Myrtol (z.B. Gelomyrtol forte)
7. Thymian/Efeu (z.B. Bronchipret)
8. Pelargonium-Extrakt (z.B. Umckaloabo)
9. Demulzentien (Honig, Hustenbonbons)
10. Hustentees
11. Phys. Kochsalz/Emser Salz Inhalation
12. Sonstige:

Sofern Sie „Sonstige“ ausgewählt haben, bitte nennen Sie die Wirkstoffe/Produkte:

_____________________________

**F4. Gründe für die Wirkstoff-/Produktempfehlung**

Warum empfehlen Sie die folgenden Wirkstoffe/Produkte im Falle eines **akuten Erkältungshusten** bevorzugt?

1. #Produkt von Platz 1 aus F3#: ________________
2. #Produkt von Platz 2 aus F3#: ________________
3. #Produkt von Platz 3 aus F3#: ________________

**F5. Wirksamkeit**

Nun geht es um die **Wirksamkeit** der einzelnen Wirkstoffe/Produkte für die Behandlung von einem **akuten Erkältungshusten.**

Welche dieser Wirkstoffe/Produkte sind Ihrer Meinung zur Symptomlinderung am **wirksamsten**?

1. N-Acetylcystein (z.B. ACC akut, ACC Generika)
2. Ambroxol (z.B. Mucosolvan, Ambroxol Generika)
3. Thymian/Primel (z.B. Bronchicum)
4. Efeu (z. B. Prospan)
5. Guaifenesin (z.B. WickHustenlöser)
6. Myrtol (z.B. Gelomyrtol forte)
7. Thymian/ Efeu (z.B. Bronchipret)
8. Pelargonium-Extrakt (z.B. Umckaloabo)
9. Demulzentien (Honig, Hustenbonbons)
10. Hustentees
11. Phys. Kochsalz/Emser Salz Inhalation
12. Sonstige: ____________

Sofern Sie „Sonstige“ ausgewählt haben: Bitte nennen Sie die Wirkstoffe/Produkte:

_____________________________

**F6. Verträglichkeit**

Nun geht es um die **Verträglichkeit** der einzelnen Wirkstoffe/Produkte für die Behandlung eines **akuten Erkältungshusten.**

Welche dieser Wirkstoffe/Produkte sind Ihrer Meinung nach zur Symptomlinderung eines akuten Erkältungshusten am **verträglichsten**?

1. N-Acetylcystein (z.B. ACC akut, ACC Generika)
2. Ambroxol (z.B. Mucosolvan, Ambroxol Generika)
3. Thymian/Primel (z.B. Bronchicum)
4. Efeu (z. B. Prospan)
5. Guaifenesin (z.B. WickHustenlöser)
6. Myrtol (z.B. Gelomyrtol forte)
7. Thymian/ Efeu (z.B. Bronchipret)
8. Pelargonium-Extrakt (z.B. Umckaloabo)
9. Demulzentien (Honig, Hustenbonbons)
10. Hustentees
11. Phys. Kochsalz/Emser Salz Inhalation
12. Sonstige

Sofern Sie „Sonstige“ ausgewählt haben: Bitte nennen Sie die Wirkstoffe/Produkte:

_____________________________

**FALLBEISPIEL 2 (NUR AN ALLGEMEINÄRZTE; FRAGEN F7-F11)**

Im Folgenden geht es um das Thema **akuter Husten** bei **akuter viraler Bronchitis** und hierbei speziell um die Bewertung ausgewählter rezeptfreier Expektorantien zur Behandlung von akutem Husten.

**Folgendes Fallbeispiel:**

Ein 60-jähriger Patient mit akutem Husten leidet unter einer **diagnostizierten akuten Bronchitis viraler Genese**. Die Anamnese/Untersuchung ergab keine weiteren Alarmzeichen. Er möchte eine symptomatische Therapie zur Linderung der Intensität und Verkürzung der Dauer seiner akuten Hustenbeschwerden.

Beantworten Sie für dieses Fallbeispiel die nachfolgenden Fragen.

**F7. Produktempfehlung bei akuter viraler Bronchitis**

Hier sehen Sie verschiedene Wirkstoffe/Produkte für die Behandlung eines **akuten Erkältungshustens** bei einem **Patienten mit akuter viraler Bronchitis.**

Welche dieser Wirkstoffe/Produkte **empfehlen** Sie dem Patienten aus dem eben beschriebenen Fallbeispiel zur Symptomlinderung bevorzugt?

1. N-Acetylcystein (z.B. ACC akut, ACC Generika)
2. Ambroxol (z.B. Mucosolvan, Ambroxol Generika)
3. Thymian/Primel (z.B. Bronchicum)
4. Efeu (z.B. Prospan)
5. Guaifenesin (z.B. WickHustenlöser)
6. Myrtol (z.B. Gelomyrtol forte)
7. Thymian/Efeu (z.B. Bronchipret)
8. Pelargonium-Extrakt (z.B. Umckaloabo)
9. Demulzentien (Honig, Hustenbonbons)
10. Hustentees
11. Phys. Kochsalz/Emser Salz Inhalation
12. Sonstige: ____________

Sofern Sie „Sonstige“ ausgewählt haben: Bitte nennen Sie die Wirkstoffe/Produkte:

_____________________________

**F8. Gründe für die Wirkstoff-/Produktempfehlung bei akutem Husten auf Grund akuter viraler Bronchitis**

Warum empfehlen Sie die folgenden Wirkstoffe/Produkte bei **akuter viraler Bronchitis** bevorzugt?

1. #Produkt von Platz 1 aus F7#: ________________
2. #Produkt von Platz 2 aus F7#: ________________
3. #Produkt von Platz 3 aus F7#: ________________

**F9. Wirksamkeit bei akuter viraler Bronchitis**

Nun geht es um die **Wirksamkeit** der einzelnen Wirkstoffe/Produkte für die Behandlung eines **akuten Erkältungshustens bei einem Patienten mit diagnostizierter akuter viraler Bronchitis.**

Welche dieser Wirkstoffe/Produkte sind Ihrer Meinung nach am **wirksamsten**?

1. N-Acetylcystein (z.B. ACC akut, ACC Generika)
2. Ambroxol (z.B. Mucosolvan, Ambroxol Generika)
3. Thymian/Primel (z.B. Bronchicum)
4. Efeu (z.B. Prospan)
5. Guaifenesin (z.B. WickHustenlöser)
6. Myrtol (z.B. Gelomyrtol forte)
7. Thymian/Efeu (z.B. Bronchipret)
8. Pelargonium-Extrakt (z.B. Umckaloabo)
9. Demulzentien (Honig, Hustenbonbons)
10. Hustentees
11. Phys. Kochsalz/Emser Salz Inhalation
12. Sonstige: ____________

Sofern Sie „Sonstige“ ausgewählt haben: Bitte nennen Sie die Wirkstoffe/Produkte:

_____________________________

**F10. Verträglichkeit bei akuter viraler Bronchitis**

Nun geht es um die **Verträglichkeit** der einzelnen Wirkstoffe/Produkte für die symptomatische Behandlung eines **akuten Erkältungshustens bei einem Patienten mit akuter viraler Bronchitis.**

Welche dieser Wirkstoffe/Produkte sind Ihrer Meinung nach am **verträglichsten**?

1. N-Acetylcystein (z.B. ACC akut, ACC Generika)
2. Ambroxol (z.B. Mucosolvan, Ambroxol Generika)
3. Thymian/Primel (z.B. Bronchicum)
4. Efeu (z.B. Prospan)
5. Guaifenesin (z.B. WickHustenlöser)
6. Myrtol (z.B. Gelomyrtol forte)
7. Thymian/Efeu (z.B. Bronchipret)
8. Pelargonium-Extrakt (z.B. Umckaloabo)
9. Demulzentien (Honig, Hustenbonbons)
10. Hustentees
11. Phys. Kochsalz/Emser Salz Inhalation
12. Sonstige: ____________

Sofern Sie „Sonstige“ ausgewählt haben: Bitte nennen Sie die Wirkstoffe/Produkte:

_____________________________

**F11. Empfehlung von Expektorantien**

Ganz allgemein: Wie häufig empfehlen Sie normalerweise Expektorantien (Sektretolytika/Mucolytika)?

*Skala: (fast) nie | selten | gelegentlich | häufig | sehr häufig*

**Zwischenseite: Datenlage**

**Im Folgenden geht es um die Datenlage** **der einzelnen Wirkstoffe**.

Mit Datenlage meinen wir hochwertige Studien, die die Wirksamkeit, Verträglichkeit und Sicherheit belegen, evidenzbasierte Leitlinien-Empfehlung, etc.

**F12. Datenlage**

Wie beurteilen Sie die folgenden Wirkstoffe hinsichtlich ihrer Datenlage?

Skala: 1 = „unzureichend“ bis 5 = „sehr gut“

1. N-Acetylcystein
2. Ambroxol
3. Myrtol
4. Efeu
5. Thymian/Primel
6. Thymian/Efeu
7. Guaifenesin

**F13a. Gründe für die gute Datenlage**

Bitte erläutern Sie, warum die **Wirksamkeit** der gewählten Wirkstoffe Ihrer Meinung nach eine besonders **gute Datenlage** aufweisen.

1. N-Acetylcystein ________________
2. Ambroxol ________________
3. Myrtol ________________
4. Efeu ________________
5. Thymian/Primel ________________
6. Thymian/Efeu ________________
7. Guaifenesin ________________

**F13b. Gründe für keine gute Datenlage**

Bitte erläutern Sie, warum die **Wirksamkeit** der gewählten Wirkstoffe Ihrer Meinung nach **keine** besonders **gute Datenlage** aufweisen.

1. N-Acetylcystein ________________
2. Ambroxol ________________
3. Myrtol ________________
4. Efeu ________________
5. Thymian/Primel ________________
6. Thymian/Efeu ________________
7. Guaifenesin ________________

**F14. Besondere zusätzliche pharmakologische Eigenschaften von Expektorantien**

Bitte denken Sie nun an besondere **pharmakologische** **Eigenschaften** von Expektorantien, wie antioxidativ, antiinflammatorisch, lokalanästhetisch, „antiviral“.

Inwieweit halten Sie die besonderen pharmakologischen Eigenschaften von Expektorantien neben der sekretolytische/mucolytischen Wirkung für eine effektive symptomatische Therapie des akuten Erkältungshustens für notwendig?

Skala: 1 = „gar nicht notwendig“ bis 5 = „äußerst notwendig“

1. Antioxidativ
2. Antiinflammatorisch
3. Lokalanästhetisch
4. Inhibitorische Effekte auf die Virus Replikation („antiviral“)

**F15. Bekanntheit Leitlinien**

Ganz allgemein, wie gut sind Ihnen die aktuellen **Leitlinien zum Thema Husten** vertraut?

1. Überhaupt nicht gut vertraut
2. …
3. …
4. …
5. Sehr gut vertraut

**F16. Hustenleitlinien**

Welche Leitlinien zum **Thema Husten** kennen Sie?

1. Bundesvereinigung Deutscher Apothekerverbände e.V. (ABDA)
2. Bundesapothekerkammer (BAK)
3. Leitlinien Deutscher Medizinischer Fachgesellschaften (z.B. DGP, DEGAM, Deutsche Atemwegsliga e.V.)
4. Leitlinien europäischer medizinischer Fachgesellschaften (z.B.Europäische Gesellschaft für Atemwegserkrankungen/ European Respiratory Society (ERS)
5. Sonstiges: _____________

**Zwischenseite: Informationstext für die Befragten**

Hier sehen Sie einen Auszug aus der neuen **Leitlinie der Deutschen Gesellschaft für Pneumologie und Beatmungsmedizin** (DGP **2019)** zur Behandlung des **akuten Erkältungshustens beim Erwachsenen.**

Bitte lesen Sie sich folgende Informationen aufmerksam durch.

**Die Neuerungen und aktuellen Empfehlungen der S2k DGP Hustenleitlinie 2019 umfassen u.a. folgende Punkte:**

- Bei ansonsten gesunden Personen mit akutem Erkältungshusten soll primär **keine antibiotische Therapie** eingeleitet werden, da die häufigste Ursache eines **akuten Erkältungshustens meist ein viraler Infekt (>50% Rhinoviren)** der oberen und/oder unteren Atemwege ist.
- Entscheidend für die **Therapieempfehlung** ist primär die **Hustendauer**, wobei der akute Husten (unter 2 Wochen) die Domäne der Selbstmedikation ist.
- **Klar empfohlen wird die symptomatische Therapie** des akuten Hustens zur Verkürzung der Dauer und Linderung der Intensität des akuten Hustens.
- Die Verordnung von **Medikamenten mit nachgewiesener Wirkung** (randomisierte klinische Studien, RCT), zur **Verkürzung der Dauer und Linderung der Intensität des akuten Hustens**.
- Empfohlen wird die Verordnung bestimmter **Phytotherapeutika** oder **Ambroxol** mit in **RCTs nachgewiesener Wirksamkeit** zwecks der Linderung der Intensität und Verkürzung der Dauer des Hustens.
- Diese **Kriterien erfüllt Ambroxol** und wird **neu** in die Leitlinienempfehlung aufgenommen.

**Diese Daten zu Wirksamkeit überzeugten die Leitlinien-Experten für ihre Therapieempfehlung:**

- Bezüglich der Wirksamkeit synthetischer Expektorantien auf den Husten bei akuter Bronchitis **gibt es nur 2 methodisch akzeptable randomisierte, placebokontrollierte Studien**: eine davon **für Ambroxol.**
- Viele **Patienten** geben eine **günstige subjektive Wirksamkeit** auf den Husten bei Selbstmedikation z. B. mit **Ambroxol** bei der akuten Bronchitis an, wie Daten einer apothekenbasierten Patientenbefragung zeigen.
- In **präklinischen Studien** zeigen Expektorantien **weitere Eigenschaften** (antientzündlich, antioxidativ, lokalanästhetisch, antiviral), die für ihre **Wirksamkeit** mitverantwortlich sein könnten.
- **Ambroxol** verfügt über diese **zusätzlichen** positiven pharmakologischen **Eigenschaften**.

**So beurteilen die Leitlinien-Experten die allgemeine Verträglichkeit der bevorzugten pflanzlichen und chemischen Expektorantien:**

- Bekannte Nebenwirkungen der gebräuchlichsten **pflanzlichen Expektorantien** sind Allergien, Hautreaktionen, Magen-Darm-Beschwerden und der Alkoholgehalt.
- Bekannte Nebenwirkungen der **chemischen Expektorantien** (Ambroxol, Bromhexin, N-Acetylcystein) sind Übelkeit und Typ IVc Hypersensitivität.
- Bei **Salzlösungen** (NaCl, Emser) zur Inhalation wird Bronchospasmus als Nebenwirkung beschrieben.

***Quelle: Aktuelle Leitlinien der Deutschen Gesellschaft für Pneumologie und Beatmungsmedizin e.V.* (https://pneumologie.de/publikationen/leitlinien/)**

**F17. Bekanntheit Inhalte der Leitlinie**

Waren Ihnen die Inhalte der neuen S2k DGP Hustenleitlinie bereits bekannt?

1. Ja, mir waren die Inhalte voll und ganz bekannt.
2. Ja, mit waren die Inhalte teilweise bekannt.
3. Nein, mir waren die Inhalte bisher nicht bekannt.

**FALLBEISPIEL 1 (F18-F21) NUR AN APOTHEKER/PTA**

**Bitte denken Sie nochmals an das Fallbeispiel:**

Eine 42-jährige Kundin klagt über **akuten Husten** bedingt durch einen **Erkältungsinfekt** mit typischer Symptomatologie ohne weitere Alarmzeichen. Es liegt keine Grunderkrankung vor. Diese Kundin kommt zu Ihnen und sucht Rat.

**F18. Produktempfehlung, Leitlinien**

Nachdem Sie die aktuell gültigen Leitlinienempfehlungen gelesen haben: Wie würden Sie persönlich - mit dem Wissen um die Evidenz - die folgenden Wirkstoffe/Produkte für die Behandlung des **akuten Erkältungshustens** der Kundin aus dem Fallbeispiel **empfehlen**?

1. N-Acetylcystein (z.B. ACC akut, ACC Generika)
2. Ambroxol (z.B. Mucosolvan, Ambroxol Generika)
3. Thymian/Primel (z.B. Bronchicum)
4. Efeu (z.B. Prospan)
5. Guaifenesin (z.B. WickHustenlöser)
6. Myrtol (z.B. Gelomyrtol forte)
7. Thymian/Efeu (z.B. Bronchipret)
8. Pelargonium-Extrakt (z.B. Umckaloabo)
9. Demulzentien (Honig, Hustenbonbons)
10. Hustentees
11. Phys. Kochsalz/Emser Salz Inhalation
12. Sonstige: ____________

Sofern Sie „Sonstige“ ausgewählt haben: Bitte nennen Sie die Wirkstoffe/Produkte:

_____________________________

**F19. Gründe für die Wirkstoff-/Produktempfehlung, Leitlinie**

Bitte erläutern Sie erneut: Warum empfehlen Sie die folgenden Wirkstoffe/Produkte bei **akutem Erkältungshusten** (nun) bevorzugt?

1. #Produkt von Platz 1 aus F18#: ________________
2. #Produkt von Platz 2 aus F18#: ________________
3. #Produkt von Platz 3 aus F18#: ________________

**F20. Wirksamkeit, Leitlinie**

Nachdem Sie die aktuell gültigen Leitlinienempfehlungen gelesen haben: Welche dieser Wirkstoffe/Produkte sind Ihrer Meinung nach am **wirksamsten** bei einem **akuten Erkältungshusten**?

1. N-Acetylcystein (z.B. ACC akut, ACC Generika)
2. Ambroxol (z.B. Mucosolvan, Ambroxol Generika)
3. Thymian/Primel (z.B. Bronchicum)
4. Efeu (z.B. Prospan)
5. Guaifenesin (z.B. WickHustenlöser)
6. Myrtol (z.B. Gelomyrtol forte)
7. Thymian/Efeu (z.B. Bronchipret)
8. Pelargonium-Extrakt (z.B. Umckaloabo)
9. Demulzentien (Honig, Hustenbonbons)
10. Hustentees
11. Phys. Kochsalz/Emser Salz Inhalation
12. Sonstige: ____________

Sofern Sie „Sonstige“ ausgewählt haben: Bitte nennen Sie die Wirkstoffe/Produkte: ____________________________

**F21. Verträglichkeit, Leitlinie**

Nachdem Sie die aktuell gültigen Leitlinienempfehlungen gelesen haben: Welche dieser Wirkstoffe/Produkte sind Ihrer Meinung nach am **verträglichsten** bei einem **akuten Erkältungshusten**?

1. N-Acetylcystein (z.B. ACC akut, ACC Generika)
2. Ambroxol (z.B. Mucosolvan, Ambroxol Generika)
3. Thymian/Primel (z.B. Bronchicum)
4. Efeu (z.B. Prospan)
5. Guaifenesin (z.B. WickHustenlöser)
6. Myrtol (z.B. Gelomyrtol forte)
7. Thymian/Efeu (z.B. Bronchipret)
8. Pelargonium-Extrakt (z.B. Umckaloabo)
9. Demulzentien (Honig, Hustenbonbons)
10. Hustentees
11. Phys. Kochsalz/Emser Salz Inhalation
12. Sonstige: ____________

Sofern Sie „Sonstige“ ausgewählt haben: Bitte nennen Sie die Wirkstoffe/Produkte:

______________________

**FALLBEISPIEL 2 (F22-F25) NUR AN ALLGEMEINÄRZTE**

**Bitte denken Sie nochmals an das Fallbeispiel:**

Ein 60-jähriger Patient mit akutem Husten leidet unter einer **diagnostizierten akuten Bronchitis viraler Genese**. Die Anamnese/Untersuchung ergab keine weiteren Alarmzeichen. Er möchte eine symptomatische Therapie zur Linderung der Intensität und Verkürzung der Dauer seiner akuten Hustenbeschwerden.

**F22. Produktempfehlung bei akuter viraler Bronchitis, Leitlinie**

Nachdem Sie die aktuell gültigen Leitlinienempfehlungen gelesen haben: Wie würden Sie persönlich - mit dem Wissen um die Evidenz - die folgenden Wirkstoffe/Produkte für die Behandlung des Patienten aus dem Fallbeispiel bei **akutem Erkältungshusten mit akuter viraler Bronchitis empfehlen**?

1. N-Acetylcystein (z.B. ACC akut, ACC Generika)
2. Ambroxol (z.B. Mucosolvan, Ambroxol Generika)
3. Thymian/Primel (z.B. Bronchicum)
4. Efeu (z.B. Prospan)
5. Guaifenesin (z.B. WickHustenlöser)
6. Myrtol (z.B. Gelomyrtol forte)
7. Thymian/Efeu (z.B. Bronchipret)
8. Pelargonium-Extrakt (z.B. Umckaloabo)
9. Demulzentien (Honig, Hustenbonbons)
10. Hustentees
11. Phys. Kochsalz/Emser Salz Inhalation
12. Sonstige: ____________

Sofern Sie „Sonstige“ ausgewählt haben: Bitte nennen Sie die Wirkstoffe/Produkte:

_____________________________

**F23. Gründe für die Wirkstoff-/Produktempfehlung bei akuter viraler Bronchitis, Leitlinie**

Bitte erläutern Sie erneut: Warum empfehlen Sie die folgenden Wirkstoffe/Produkte bei **akuter viraler Bronchitis** (nun) bevorzugt?

1. #Produkt von Platz 1 aus F22#: ________________
2. #Produkt von Platz 2 aus F22#: ________________
3. #Produkt von Platz 3 aus F22#: ________________

**F24. Wirksamkeit bei akuter viraler Bronchitis, Leitlinie**

Nachdem Sie die aktuell gültigen Leitlinienempfehlungen gelesen haben: Welche dieser Wirkstoffe/Produkte sind Ihrer Meinung nach am **wirksamsten** bei einem **akuten Erkältungshusten mit diagnostizierter akuter viraler Bronchitis**?

1. N-Acetylcystein (z.B. ACC akut, ACC Generika)
2. Ambroxol (z.B. Mucosolvan, Ambroxol Generika)
3. Thymian/Primel (z.B. Bronchicum)
4. Efeu (z.B. Prospan)
5. Guaifenesin (z.B. WickHustenlöser)
6. Myrtol (z.B. Gelomyrtol forte)
7. Thymian/Efeu (z.B. Bronchipret)
8. Pelargonium-Extrakt (z.B. Umckaloabo)
9. Demulzentien (Honig, Hustenbonbons)
10. Hustentees
11. Phys. Kochsalz/Emser Salz Inhalation
12. Sonstige: ____________

Sofern Sie „Sonstige“ ausgewählt haben: Bitte nennen Sie die Wirkstoffe/Produkte:

_____________________________

**F25. Verträglichkeit bei akuter viraler Bronchitis, Leitlinie**

Nachdem Sie die aktuell gültigen Leitlinienempfehlungen gelesen haben: Welche dieser Wirkstoffe/Produkte sind Ihrer Meinung nach am **verträglichsten** bei einem **akuten Erkältungshusten mit akuter viraler Bronchitis**?

1. N-Acetylcystein (z.B. ACC akut, ACC Generika)
2. Ambroxol (z.B. Mucosolvan, Ambroxol Generika)
3. Thymian/Primel (z.B. Bronchicum)
4. Efeu (z.B. Prospan)
5. Guaifenesin (z.B. WickHustenlöser)
6. Myrtol (z.B. Gelomyrtol forte)
7. Thymian/Efeu (z.B. Bronchipret)
8. Pelargonium-Extrakt (z.B. Umckaloabo)
9. Demulzentien (Honig, Hustenbonbons)
10. Hustentees
11. Phys. Kochsalz/Emser Salz Inhalation
12. Sonstige: ____________

Sofern Sie „Sonstige“ ausgewählt haben: Bitte nennen Sie die Wirkstoffe/Produkte:

_____________________________

**Zwischenseite Datenlage, Leitlinie**

**Im Folgenden geht es noch einmal um die** **Datenlage der einzelnen Wirkstoffe**. Mit Datenlage meinen wir hochwertige Studien, die die Wirksamkeit, Verträglichkeit und Sicherheit belegen, evidenzbasierte Leitlinien-Empfehlung, etc.

**F26. Datenlage, Leitlinie**

Wie beurteilen Sie nun die folgenden Wirkstoffe hinsichtlich Ihrer Datenlage?

Skala: 1 = „unzureichend“ bis 5 = „sehr gut“

1. N-Acetylcystein
2. Ambroxol
3. Myrtol
4. Efeu
5. Thymian/Primel
6. Thymian/Efeu
7. Guaifenesin

**F27a. Gründe für die gute Datenlage, Leitlinie**

Bitte erläutern Sie, warum die **Wirksamkeit** der gewählten Wirkstoffe Ihrer Meinung nach eine besonders **gute Datenlage** aufweisen.

1. N-Acetylcystein ________________
2. Ambroxol ________________
3. Myrtol ________________
4. Efeu ________________
5. Thymian/Primel ________________
6. Thymian/Efeu ________________
7. Guaifenesin ________________

**F27b. Gründe für keine gute Datenlage, Leitlinie**

Bitte erläutern Sie, warum die **Wirksamkeit** der gewählten Wirkstoffe Ihrer Meinung nach **keine** besonders **gute Datenlage** aufweisen.

1. N-Acetylcystein ________________
2. Ambroxol ________________
3. Myrtol ________________
4. Efeu ________________
5. Thymian/Primel ________________
6. Thymian/Efeu ________________
7. Guaifenesin ________________

**F28. Besondere zusätzliche pharmakologische Eigenschaften von Expektorantien, Leitlinie**

Bitte denken Sie nun auch noch einmal an die besonderen **pharmakologischen** **Eigenschaften** von Expektorantien, wie antioxidativ, antiinflammatorisch, lokalanästhetisch, „antiviral“.

Inwieweit halten Sie die besonderen pharmakologischen Eigenschaften von Expektorantien neben der sekretolytische/mucolytischen Wirkung für eine effektive symptomatische Therapie des akuten Erkältungshustens für notwendig?

Skala: 1 = „gar nicht notwendig“ bis 5 = „äußerst notwendig“

1. Antioxidativ
2. Antiinflammatorisch
3. Lokalanästhetisch
4. Inhibitorische Effekte auf die Virus Replikation („antiviral“)

**F29. Eigenschaften von Wirkstoffen**

Hier sehen Sie verschiedene Aussagen zum Thema “besondere pharmakologische Eigenschaften“. Welche dieser Aussagen trifft Ihrer Ansicht nach auf **Ambroxol** zu?

*Bitte wählen Sie alle Antworten, die für Sie zutreffen.*

Ambroxol…

1. …stimuliert die Aktivität des Flimmerepithels
2. …steigert die Surfactant-Produktion im Lungengewebe
3. …führt bei COPD Patienten zu einer Reduktion von Exazerbationen
4. …hat lokalanästhetische Effekte
5. …zeigt *in vitro* antiinflammatorische, antioxidative Eigenschaften
6. …führt zu einer Anreicherung von Antibiotika (z.B. Amoxicillin) im Sputum und Bronchialsekret

7) …zeigt *in vitro* eine Verminderung der Replikation von Rhinoviren

**SOZIODEMOGRAPHIE**

**nur Apotheker und PTAs**

**SO7. Apothekengröße**

Nun noch wenige Fragen für die Statistik.

Wie viele Verkaufspunkte hat die Apotheke, in der Sie beschäftigt sind?

1. 1-2 Verkaufspunkte
2. 3-4 Verkaufspunkte
3. 5 oder mehr Verkaufspunkte

**Filter: nur Ärzte**

### SO8. Praxisgröße

Nun noch wenige Fragen für die Statistik.

Wie viele Scheine rechnen Sie im Schnitt pro Quartal ab?

1. unter 600
2. 600 bis unter 800
3. 800 bis unter 1000
4. 1000 bis unter 1200
5. 1200 bis unter 1400
6. 1400 und mehr

**SO9. Ortsgröße**

Wie viele Einwohner hat der Ort, in dem Sie tätig sind?

1. Weniger als 5.000 (Landstadt)
2. 5.000 bis unter 20.000 (Kleinstadt)
3. 20.000 bis unter 100.000 (Mittelstadt)
4. 100.000 bis unter 1 Mio. (Großstadt)
5. ab 1 Mio. (Millionenstadt)

**SO10. Geschlecht**

Sind Sie…?

1. Männlich
2. Weiblich
3. Divers

**SO11. Alter**

Wie alt sind Sie?

_______Jahre

**English translation of questionnaire**

**Q1. Information seeking behavior**

The initial topic is information. Generally, how often do you specifically seek information on **the topic of** **cough/bronchial** **disease**?

*Scale: never | rarely | sometimes | often | very often*

**Q2. Sources of information**

Which sources do you normally use to obtain information on **the topic of cough/bronchial disease**?

*Please select all applicable answers.*

1. publications by medical associations
2. professional journals
3. treatment guidelines
4. professional information in Internet
5. conversations with sales representatives
6. trainings by pharmaceutical companies
7. trainings by board of physicians/pharmacists
8. continuing education by board of physicians/pharmacists
9. trainings by professional organizations
10. professional lectures
11. reports on TV, radio
12. conversations with customers/patients
13. internet (e.g., Google/ Wikipedia)
14. other:_______________

**Case 1 (Q3-Q6) ONLY FOR PHARMACISTS/PHARMACY TECHNICIANS**

Hereafter we focus on the topic of **acute cough associated with a common cold**, specifically your judgement of selected, prescription-free expectorants for the treatment of acute cough.

**Case:**

A 42-year-old, female customer complains about **acute cough** caused by a **common cold** with typical symptoms and without alarm symptoms. There is no underlying disease. This customer asks for your advice.

Answer the following questions as applicable to this case.

**Q3. Product recommendation**

Here you see various active ingredients/products for the treatment of acute **common cold**. Which of these active ingredients/products do you recommend for relief of cough in the case describe above as preferred?

1. N-acetylcysteine (e.g., ACC akut, ACC generics)
2. ambroxol (e.g., Mucosolvan, ambroxol generics)
3. thyme/primrose (e.g., Bronchicum)
4. ivy (e.g., Prospan)
5. guaifenesin (e.g., WickHustenlöser)
6. myrtol (e.g., Gelomyrtol forte)
7. thyme/ivy (e.g., Bronchipret)
8. pelargonium extract (e.g., Umckaloabo)
9. demulcents (honey, cough drops)
10. cough remedy teas
11. physiological saline/Emser salt inhalation
12. other:

If ‚other‘, please specify active ingredient/product:

_____________________________

**Q4. Reasons for recommendation of active ingredient/product**

Why do you recommend the following active ingredient/product in case of **acute cough associated with common cold**?

1. First ranked product of F3#: ________________
2. Second ranked product of F3#: ________________
3. Third ranked product of F3#: ________________

**Q5. Efficacy**

We now talk about **efficacy** of the active ingredients/products for the treatment of **acute cough associated with common cold.**

Which of these active ingredients/products are **most effective** in your opinion?

1. N-acetylcysteine (e.g., ACC akut, ACC generics)
2. ambroxol (e.g., Mucosolvan, ambroxol generics)
3. thyme/primrose (e.g., Bronchicum)
4. ivy (e.g., Prospan)
5. guaifenesin (e.g., WickHustenlöser)
6. myrtol (e.g., Gelomyrtol forte)
7. thyme/ivy (e.g., Bronchipret)
8. pelargonium extract (e.g., Umckaloabo)
9. demulcents (honey, cough drops)
10. cough remedy teas
11. physiological saline/Emser salt inhalation
12. other:

If ‚other‘, please specify active ingredient/product:

_____________________________

**Q6. Tolerability**

We now talk about **tolerability** of the various active ingredients/products for the treatment of **acute cough associated with common cold.**

Which of these active ingredients/products are **best tolerated** in the alleviation of symptoms of acute cough associated with common cold?

1. N-acetylcysteine (e.g., ACC akut, ACC generics)
2. ambroxol (e.g., Mucosolvan, ambroxol generics)
3. thyme/primrose (e.g., Bronchicum)
4. ivy (e.g., Prospan)
5. guaifenesin (e.g., WickHustenlöser)
6. myrtol (e.g., Gelomyrtol forte)
7. thyme/ivy (e.g., Bronchipret)
8. pelargonium extract (e.g., Umckaloabo)
9. demulcents (honey, cough drops)
10. cough remedy teas
11. physiological saline/Emser salt inhalation
12. other:

If ‚other‘, please specify active ingredient/product:

_____________________________

**CASE 2 (Q7-Q11) ONLY FOR GENERAL PRACTITIONERS**

Hereafter we focus on the topic of **acute cough** during **acute viral bronchitis** and specifically your judgment of selected, prescription-free expectorants for the treatment of acute cough.

**Case:**

A 60-year-old male patient with acute cough suffers from a **diagnosed acute bronchitis of viral origin**. The history and physical examination yielded no alarm symptoms. He wishes to receive a symptomatic treatment for the alleviation of the intensity and shortening of the duration of his acute cough complaints.

Please answer the following questions for this case.

**Q7. Product recommendation for acute viral bronchitis**

Here you see various active ingredients/products for the treatment of acute **cough** in a **patient with acute viral bronchitis**. Which of these active ingredients/products do you recommend for relief of cough in the case describe above as preferred?

1. N-acetylcysteine (e.g., ACC akut, ACC generics)
2. ambroxol (e.g., Mucosolvan, ambroxol generics)
3. thyme/primrose (e.g., Bronchicum)
4. ivy (e.g., Prospan)
5. guaifenesin (e.g., WickHustenlöser)
6. myrtol (e.g., Gelomyrtol forte)
7. thyme/ivy (e.g., Bronchipret)
8. pelargonium extract (e.g., Umckaloabo)
9. demulcents (honey, cough drops)
10. cough remedy teas
11. physiological saline/Emser salt inhalation
12. other:

If ‚other‘, please specify active ingredient/product:

_____________________________

**Q8. Reasons for recommendation of active ingredient/product for acute cough in acute viral bronchitis**

Why do you recommend the following active ingredients/products in **acute virale bronchitis**?

1. First ranked product of Q7#: ________________
2. Second ranked product of Q7#: ________________
3. Third ranked product of Q7#: ________________

**Q9. Efficacy in acute viral bronchitis**

We now talk about the **efficacy** of the various active ingredients/products in the treatment of **acute cough in a patient with diagnosed acute viral bronchitis.**

Which of these active ingredients/products is most **efficacious** in your opinion?

1. N-acetylcysteine (e.g., ACC akut, ACC generics)
2. ambroxol (e.g., Mucosolvan, ambroxol generics)
3. thyme/primrose (e.g., Bronchicum)
4. ivy (e.g., Prospan)
5. guaifenesin (e.g., WickHustenlöser)
6. myrtol (e.g., Gelomyrtol forte)
7. thyme/ivy (e.g., Bronchipret)
8. pelargonium extract (e.g., Umckaloabo)
9. demulcents (honey, cough drops)
10. cough remedy teas
11. physiological saline/Emser salt inhalation
12. other:

If ‚other‘, please specify active ingredient/product:

_____________________________

**Q10. Tolerability in acute viral bronchitis**

We now talk about the **tolerability** of the active ingredients/products for the symptomatic treatment of **acute cough in a patient with acute viral bronchitis.**

Which of these active ingredients/products is ***best tolerated*** in your opinion?

1. N-acetylcysteine (e.g., ACC akut, ACC generics)
2. ambroxol (e.g., Mucosolvan, ambroxol generics)
3. thyme/primrose (e.g., Bronchicum)
4. ivy (e.g., Prospan)
5. guaifenesin (e.g., WickHustenlöser)
6. myrtol (e.g., Gelomyrtol forte)
7. thyme/ivy (e.g., Bronchipret)
8. pelargonium extract (e.g., Umckaloabo)
9. demulcents (honey, cough drops)
10. cough remedy teas
11. physiological saline/Emser salt inhalation
12. other:

If ‚other‘, please specify active ingredient/product:

_____________________________

**Q11. Recommendation of expectorants**

Generally, how often do you normally recommend expectorants (secretolytics/mucolytics)?

*Scale: (almost) never | rarely | sometimes | frequently | very frequently*

**Intermediate page: evidence base**

**We now talk about the evidence base of the active ingredients**.

‘Evidence base‘ here means studies of high quality, that support efficacy, tolerability and safety, recommendations from evidence-based guidelines, etc.

**Q12. Evidence base**

How do you rate the following active ingredients for their evidence base?

Scale: 1 = „insufficient “ to 5 = „very good “

1. N-acetylcysteine
2. ambroxol
3. myrtol
4. ivy
5. thyme/primrose
6. thyme/ivy
7. guaifenesin

**Q13a. Reasons for good evidence base**

Please explain why the selective active ingredients have a **good evidence base** for **efficacy**.

1. N-acetylcysteine ________________
2. ambroxol ________________
3. myrtol ________________
4. ivy ________________
5. thyme/primrose ________________
6. thyme/ivy ________________
7. guaifenesin ________________

**Q13b. Reasons for lack of good evidence base**

Please explain why the selected active ingredients **lack a good evidence base** for **efficacy**.

1. N-acetylcysteine ________________
2. ambroxol ________________
3. myrtol ________________
4. ivy ________________
5. thyme/primrose ________________
6. thyme/ivy ________________
7. guaifenesin ________________

**Q14. Special additional pharmacological properties of expectorants**

Please think now of special **pharmacological properties** of expectorants such as anti-oxidative, anti-inflammatory, local anesthetic, ‘anti-viral’.

How much do you consider special pharmacological properties of expectorants next to their secretolytic/mucolytic effect to be necessary for an effect symptomatic treatment of acute cough associated with common cold?

Scale: 1 = „not at all“ to 5 = „extremely necessary “

1. anti-oxidant
2. anti-inflammatory
3. local anesthetic
4. inhibitory effects on virus replication („anti-viral“)

**Q15. Familiarity with guidelines**

Generally, how familiar are you with current **guidelines related to cough**?

1. not familiar at all
2. …
3. …
4. …
5. very familiar

**Q16. Cough guidelines**

Which guidelines on the **topic of cough** do you know?

1. Bundesvereinigung Deutscher Apothekerverbände e.V. (ABDA)
2. Bundesapothekerkammer (BAK)
3. Guidelines of German medical societies (e.g., DGP, DEGAM, Deutsche Atemwegsliga e.V.)
4. Guidelines of European medical societies (e.g., European Respiratory Society)
5. Other: _____________

**Intermediate page: information text for participants**

You see here excerpts from the new **guidline of the Deutschen Gesellschaft für Pneumologie und Beatmungsmedizin** (DGP **2019)** for the treatment of **acute cough associated with common cold in adults.**

Please read the following information attentively.

**The updates and current recommendations of the S2k DGP cough guidelines 2019 include amongst others the following points:**

- **No antibiotic treatment** shall be initiated in otherwise healthy people with acute cough associated with common cold because the most frequent **cause of cough associated with common cold is a viral infection (>50% Rhinoviruses)** of the upper and/or lower airways.
- Decisive for the **therapeutic recommendation** is primarily the **duration of cough**, with acute cough (less than 2 weeks) being a domain of self-medication.
- **Symptomatic treatment** of acute cough **is clearly recommended** to shorten duration and relieve intensity of acute cough.
- The prescription of **medicines with proven effects** (randomized clinical trials, RCT) for the **shortening of duration and relief of intensity of acute cough**.
- Prescription of certain **phytotherapeutics** or **ambroxol** with **efficacy proven in RCTs** is recommended to relieve intensity and shorten duration of cough.
- These **criteria are fulfilled by ambroxol**, which is **newly** added to the guideline recommendation.

**These efficacy data convinced the guideline experts for their treatment recommendation:**

- **Only 2 methodologically acceptable, randomized, placebo-controlled studies** exist to support the efficacy of synthetic expectorants for cough in acute bronchitis: one of them for **ambroxol.**
- Many **patients** report a **beneficial subjective efficacy** against cough in self-medication e.g., with **ambroxol** in acute bronchitis as shown by data from a pharmacy-based patient survey.
- Experctorants exhibited **additional properties** (anti-inflammatory, anti-oxidative, local anesthetic, anti-viral) In **preclinical studies**, which could contribute to their **efficacy**.
- **Ambroxol** has these **additional** positive pharmacological **properties**.

**This is how the guideline experts judged the general tolerability of the preferred plant-derived and chemical expectorants:**

- Known side effects of the most frequently used **plant-derived expectorants** are allergies, skin reactions, stomach and gut complaints and the alcohol content.
- Know side effects of the **chemical expectorants** (ambroxol, bromohexin, N-acetylcystein) are nausea and type IVc hypersensitivity.
- Bronchospams has been reported as side effect of **salt solutions** (NaCl, Emser) for inhalation.

***Source: current guidelines of the Deutsche Gesellschaft für Pneumologie und Beatmungsmedizin e.V.* (https://pneumologie.de/publikationen/leitlinien/)**

**Q17. Familiarity with the content of the guideline**

Was the content of the new S2k DGP cough guideline already known?

1. Yes, I fully knew the content.
2. Yes, I partly knew the content.
3. No, the content was not known to me.

**CASE 1 (Q18-Q21) ONLY FOR PHARMACISTS/PHARMACY TECHNICIANS**

**Please think against of the case:**

A 42-year-old, female customer complains about **acute cough** caused by a **common cold** with typical symptoms and without alarm symptoms. There is no underlying disease. This customer asks for your advice.

**Q18. Product recommendation, guidelines**

After having read current guideline recommendations: How would you personally – knowing the evidence – **recommend** the following active ingredients/products for the treatment of **acute cough associated with common cold** to the customer from the case?

1. N-acetylcysteine (e.g., ACC akut, ACC generics)
2. ambroxol (e.g., Mucosolvan, ambroxol generics)
3. thyme/primrose (e.g., Bronchicum)
4. ivy (e.g., Prospan)
5. guaifenesin (e.g., WickHustenlöser)
6. myrtol (e.g., Gelomyrtol forte)
7. thyme/ivy (e.g., Bronchipret)
8. pelargonium extract (e.g., Umckaloabo)
9. demulcents (honey, cough drops)
10. cough remedy teas
11. physiological saline/Emser salt inhalation
12. other:

If ‚other‘, please specify active ingredient/product:

_____________________________

**Q19. Reasons for recommendation of active ingredient/product, guideline**

Please explain again: Why do you (now) preferentially recommend the following active ingredient/product in case of **acute cough associated with common cold**?

1. First ranked product of Q18#: ________________
2. Second ranked product of Q18#: ________________
3. Third ranked product of Q18#: ________________

**Q20. Efficacy, guideline**

After having read the current guideline recommendations: which of these active ingredients/products are in your opinion **most effective** in **acute cough associated with common cold**?

1. N-acetylcysteine (e.g., ACC akut, ACC generics)
2. ambroxol (e.g., Mucosolvan, ambroxol generics)
3. thyme/primrose (e.g., Bronchicum)
4. ivy (e.g., Prospan)
5. guaifenesin (e.g., WickHustenlöser)
6. myrtol (e.g., Gelomyrtol forte)
7. thyme/ivy (e.g., Bronchipret)
8. pelargonium extract (e.g., Umckaloabo)
9. demulcents (honey, cough drops)
10. cough remedy teas
11. physiological saline/Emser salt inhalation
12. other:

If ‚other‘, please specify active ingredient/product:

_____________________________

**Q21. Tolerability, guideline**

After having read the current guideline recommendations: which of these active ingredients/products are in your opinion **best tolerated** in **acute cough associated with common cold**?

1. N-acetylcysteine (e.g., ACC akut, ACC generics)
2. ambroxol (e.g., Mucosolvan, ambroxol generics)
3. thyme/primrose (e.g., Bronchicum)
4. ivy (e.g., Prospan)
5. guaifenesin (e.g., WickHustenlöser)
6. myrtol (e.g., Gelomyrtol forte)
7. thyme/ivy (e.g., Bronchipret)
8. pelargonium extract (e.g., Umckaloabo)
9. demulcents (honey, cough drops)
10. cough remedy teas
11. physiological saline/Emser salt inhalation
12. other:

If ‚other‘, please specify active ingredient/product:

_____________________________

**CASE 2 (Q22-Q25) ONLY FOR GENERAL PRACTIONERS**

**Please think again of the case:**

A 60-year-old male patient with acute cough suffers from a **diagnosed acute bronchitis of viral origin**. The history and physical examination yielded no alarm symptoms. He wishes to receive a symptomatic treatment for the alleviation of the intensity and shortening of the duration of his acute cough complaints.

**Q22. Product recommendation for acute viral bronchitis, guideline**

After having read the current guideline recommendations: How would you personally – knowing the evidence – **recommend** the following active ingredients/products for the treatment of **cough with acute viral bronchitis** to the patient from the case?

1. N-acetylcysteine (e.g., ACC akut, ACC generics)
2. ambroxol (e.g., Mucosolvan, ambroxol generics)
3. thyme/primrose (e.g., Bronchicum)
4. ivy (e.g., Prospan)
5. guaifenesin (e.g., WickHustenlöser)
6. myrtol (e.g., Gelomyrtol forte)
7. thyme/ivy (e.g., Bronchipret)
8. pelargonium extract (e.g., Umckaloabo)
9. demulcents (honey, cough drops)
10. cough remedy teas
11. physiological saline/Emser salt inhalation
12. other:

If ‚other‘, please specify active ingredient/product:

_____________________________

**Q23. Reasons for recommendation of active ingredient/product, guideline**

Please explain again: Why do you (now) preferentially recommend the following active ingredient/product in case of **acute viral bronchitis**?

1. First ranked product of Q22#: ________________
2. Second ranked product of Q22#: ________________
3. Third ranked product of Q22#: ________________

**Q24. Efficacy in acute viral bronchitis, guideline**

After having read the current guideline recommendations: which of these active ingredients/products are in your opinion **most effective** in **acute cough with diagnosed acute viral bronchitis**?

1. N-acetylcysteine (e.g., ACC akut, ACC generics)
2. ambroxol (e.g., Mucosolvan, ambroxol generics)
3. thyme/primrose (e.g., Bronchicum)
4. ivy (e.g., Prospan)
5. guaifenesin (e.g., WickHustenlöser)
6. myrtol (e.g., Gelomyrtol forte)
7. thyme/ivy (e.g., Bronchipret)
8. pelargonium extract (e.g., Umckaloabo)
9. demulcents (honey, cough drops)
10. cough remedy teas
11. physiological saline/Emser salt inhalation
12. other:

If ‚other‘, please specify active ingredient/product:

_____________________________

**Q25. Tolerability in acute viral bronchitis, guideline**

After having read the current guideline recommendations: which of these active ingredients/products are in your opinion **best tolerated** in **acute cough with acute viral bronchitis**?

1. N-acetylcysteine (e.g., ACC akut, ACC generics)
2. ambroxol (e.g., Mucosolvan, ambroxol generics)
3. thyme/primrose (e.g., Bronchicum)
4. ivy (e.g., Prospan)
5. guaifenesin (e.g., WickHustenlöser)
6. myrtol (e.g., Gelomyrtol forte)
7. thyme/ivy (e.g., Bronchipret)
8. pelargonium extract (e.g., Umckaloabo)
9. demulcents (honey, cough drops)
10. cough remedy teas
11. physiological saline/Emser salt inhalation
12. other:

If ‚other‘, please specify active ingredient/product:

_____________________________

**Intermediate page: evidence base, guideline**

**The following deals again with the evidence base of the active ingredients**. ‘Evidence base‘ here means studies of high quality, that support efficacy, tolerability and safety, recommendations from evidence-based guidelines, etc.

**Q26. Evidence base, guidelines**

How do you rate the following active ingredients for their evidence base?

Scale: 1 = „insufficient “ to 5 = „very good “

1. N-acetylcysteine
2. ambroxol
3. myrtol
4. ivy
5. thyme/primrose
6. thyme/ivy
7. guaifenesin

**Q27a. Reasons for good evidence base, guideline**

Please explain why the selective active ingredients have a **good evidence base** for **efficacy**.

1. N-acetylcysteine ________________
2. ambroxol ________________
3. myrtol ________________
4. ivy ________________
5. thyme/primrose ________________
6. thyme/ivy ________________
7. guaifenesin ________________

**Q27b. Reasons for lack of good evidence base**

Please explain why the selected active ingredients **lack a good evidence base** for **efficacy**

1. N-acetylcysteine ________________
2. ambroxol ________________
3. myrtol ________________
4. ivy ________________
5. thyme/primrose ________________
6. thyme/ivy ________________
7. guaifenesin ________________

**Q28. Special additional pharmacological properties of expectorants, guideline**

Please think again of special **pharmacological properties** of expectorants such as anti-oxidative, anti-inflammatory, local anesthetic, ‘anti-viral’.

How much do you consider special pharmacological properties of expectorants next to their secretolytic/mucolytic effect to be necessary for an effect symptomatic treatment of acute cough associated with common cold?

Scale: 1 = „not at all“ to 5 = „extremely necessary “

1. anti-oxidant
2. anti-inflammatory
3. local anesthetic
4. inhibitory effects on virus replication („anti-viral“)

**Q29. Properties of active ingredients**

Below you see several statements on the topic of „special pharmacological properties‘. Which of them in your opinion applies to **ambroxol**?

*Please select all applicable answers.*

Ambroxol…

1. …stimulates the activity of the ciliated epithelium
2. …increases the production of surfactant in lung tissue
3. …leads to a reduction of exacerbations in COPD patients
4. …has local anesthetic properties
5. …exhibits anti-inflammatory and anti-oxidative properties *in vitro*
6. …leads to enrichment of antibiotics (e.g., amoxicillin) in sputum and bronchial secretion

7) …exhibits a reduced replication of rhino viruses *in vitro*

**SOCIODEMOGRAPHICS**

**ONLY FOR PHARMACISTS/PHARMACY TECHNICIANS**

**SO7. Size of pharmacy**

A few more questions on statistics.

How many sales points has the pharmacy in which you work?

1. 1-2
2. 3-4
3. 5 or more

**ONLY FOR GENERAL PRACTIONERS**

### SO8. Size of office

A few more questions on statistics.

How many patients do you typically have per quarter?

1. Less than 600
2. 600 to 800
3. 800 to 1000
4. 1000 to 1200
5. 1200 to 1400
6. 1400 and more

**SO9. Size of town**

How many inhabitants has the town in which you work?

1. Less than 5,000 (country village)
2. 5,000 to 20,000 (small town)
3. 20,000 to 100,000 (mid-size town)
4. 100,000 to 1 Mio. (large town)
5. More than 1 Mio. (metropolitan area)

**SO10. Gender**

Are you …?

1. Male
2. Female
3. Other

**SO11. Age**

How old are you?

_______years
